# Arrdc4‐dependent extracellular vesicle biogenesis is required for sperm maturation

**DOI:** 10.1002/jev2.12113

**Published:** 2021-06-22

**Authors:** Natalie J. Foot, Macarena B. Gonzalez, Kelly Gembus, Pamali Fonseka, Jarrod J. Sandow, Thuy Tien Nguyen, Diana Tran, Andrew I. Webb, Suresh Mathivanan, Rebecca L. Robker, Sharad Kumar

**Affiliations:** ^1^ Centre for Cancer Biology University of South Australia and SA Pathology Adelaide South Australia Australia; ^2^ School of Medicine Robinson Research Institute University of Adelaide Adelaide South Australia Australia; ^3^ Department of Biochemistry and Genetics La Trobe Institute for Molecular Sciences La Trobe University Melbourne Victoria Australia; ^4^ Advanced Technology and Biology Division Walter and Eliza Hall Institute Parkville Victoria Australia; ^5^ Department of Medical Biology University of Melbourne Parkville VIC Australia; ^6^ School of Biological Sciences University of Adelaide Adelaide South Australia Australia; ^7^ School of Chemical Engineering & Advanced Materials University of Adelaide Adelaide South Australia Australia; ^8^ Department of Anatomy and Developmental Biology Biomedicine Discovery Institute Monash University Melbourne Victoria Australia; ^9^ Faculty of Health and Medical Sciences University of Adelaide Adelaide South Australia Australia

**Keywords:** arrestin, biogenesis, extracellular vesicles, fertility, sperm maturation, ubiquitin ligase adaptors

## Abstract

Extracellular vesicles (EVs) are important players in cell to cell communication in reproductive systems. Notably, EVs have been found and characterized in the male reproductive tract, however, direct functional evidence for their importance in mediating sperm function is lacking. We have previously demonstrated that Arrdc4, a member of the α‐arrestin protein family, is involved in extracellular vesicle biogenesis and release. Here we show that Arrdc4‐mediated extracellular vesicle biogenesis is required for proper sperm function. Sperm from *Arrdc4^–/–^* mice develop normally through the testis but fail to acquire adequate motility and fertilization capabilities through the epididymis, as observed by reduced motility, premature acrosome reaction, reduction in zona pellucida binding and two‐cell embryo production. We found a significant reduction in extracellular vesicle production by *Arrdc4^–/‐^* epididymal epithelial cells, and further, supplementation of *Arrdc4^–/–^* sperm with additional vesicles dampened the acrosome reaction defect and restored zona pellucida binding. These results indicate that Arrdc4 is important for proper sperm maturation through the control of extracellular vesicle biogenesis.

## 1 INTRODUCTION

Following their production in the testis, sperm acquire their functional maturity as they progress through the epididymis, a highly convoluted and specialized duct distal to the testis. It is generally accepted that due to their small size, compact nature of the nucleus, and the lack of specific organelles, sperm are unable to perform de novo protein synthesis, and thus, rely on extrinsic signals delivered to the sperm from the epididymal milieu as they travel from the proximal epididymal segments (initial segment, caput, and corpus) through to the distal storage segment (cauda). This is evident in the vast changes that take place in the sperm proteome as they travel through the epididymis, and this occurs in both mammalian and non‐mammalian species (Amaral et al., 2014; Chauvin et al., 2012; Ijiri et al., 2011; Nixon, Johnston et al., 2018; Skerget et al., 2015). Extracellular vesicles (EVs) are a major component of the epididymal luminal fluid, and are believed to be a key contributor to epididymal sperm maturation, although the mechanism is only partially understood (Girouard et al., 2011; Nixon, De Iuliis et al., 2018; Sullivan et al., 2005; Zhou et al, 2018).

EVs are small membrane‐bound vesicles released by cells that contain a variety of proteins, lipids, and genetic material that can be transferred to a recipient cell (Mathieu et al., 2019). EVs can be classified into two main sub‐types: exosomes are small EVs (30–150 nm) that are released into the extracellular microenvironment upon the fusion of multivesicular bodies with the plasma membrane, whereas microvesicles are generally larger (100‐1000 nm) and bud from directly from the plasma membrane (Mathieu et al., 2019). EVs are important in normal physiological processes (e.g., immune signalling and development), as well as virus egress and disorders such as cancer, cardiovascular disease, and inflammation (Kalluri & LeBleu, 2020). Recently there have been a number of studies demonstrating a role for EVs in tissue regeneration and repair (Liu et al., 2020; Qiao et al., 2019; Wang et al., 2020) as well as their potential as drug delivery systems (Forterre et al., 2020; Xue et al., 2020). While vesicular structures were first described in human semen over 40 years ago (Ronquist et al, 1978; Ronquist, Brody, Gottfries & Stegmayr, 1978), the physiological role of EVs in sperm function is only beginning to be elucidated. Epididymal EVs (also known as epididymosomes) are a heterogeneous population of vesicles with different sizes and cargoes. Epididymosomes from multiple species contain a vast array of proteins (Girouard et al., 2011; Nixon et al., 2019), and a few of these have now been functionally characterized. For example, CD9‐positive vesicles extracted from epididymal fluid were shown to be transferred preferentially to live sperm, whereas those extracted from blood did not (Caballero et al., 2013). Epididymal sperm binding protein 1 (ELSPBP1) is transferred by epididymosomes to dead sperm only, suggestive of a quality control mechanism which may function to either protect viable sperm or to target defective ones for degradative pathways (D'Amours et al., 2012). Members of the *C*ysteine‐rich secretory proteins, *A*ntigen 5, and *P*athogenesis‐related 1 (CAP) superfamily of sperm‐coating glycoproteins are transferred to sperm via epididymosomes, and have been proposed to be involved in capacitation‐associated events (Nixon et al., 2006; Roberts et al., 2007) and in sperm binding to the zona pellucida and plasma membrane of the oocyte (Caballero et al, 2012; Cohen et al, 2007; Cohen et al., 2008; Da Ros et al., 2007, 2008). Sperm motility is also controlled by proteins transferred by epididymosomes, such as Wnt signalling proteins (Koch et al., 2015) and ß‐defensins (Zhang et al., 2018; Zhao et al., 2004, 2011).

We and others have identified specific members of the α‐arrestin protein family as key regulators of EV release, namely Arrdc1 (Anand et al., 2018; Nabhan et al., 2012) and Arrdc4 (Mackenzie et al., 2016). α‐arrestins act as adaptors for ubiquitin ligases of the Nedd4 family (Dores et al., 2015; Foot et al., 2017; Han et al., 2013; Nabhan et al., 2010, Shah and Kumar, 2021; Shea et al., 2012). Like β‐ and visual arrestins, α‐arrestins also contain arrestin N and C domains (Alvarez, 2008), however, only the α‐arrestins (Arrdc1‐4 and TXNIP) contain C‐terminal PY motifs which bind the WW domains of the Nedd4 family of ubiquitin ligases (Nabhan et al., 2010; Qi et al., 2014; Shea et al., 2012). Arrdc1 has been implicated in the formation of EVs in a process requiring the ESCRT components Tsg101 and VPS4, and the Nedd4 family member WWP2 (Nabhan et al., 2012; Rauch & Martin‐Serrano, 2011). Arrdc4 is also important for EV formation, with loss of Arrdc4 resulting in a reduction of EV production in a number of different cell types, however it seems to rely on a different mechanism involving the recycling pathway component Rab11a (Mackenzie et al., 2016). Arrdc4 also appears to mediate the transfer of specific cargoes into EVs, such as the divalent metal ion transporter DMT1 (Mackenzie et al., 2016).

Here, we show that Arrdc4 is required for proper sperm maturation in the epididymis. Sperm from *Arrdc4^–/–^* mice develop normally through the testis but fail to acquire adequate motility and fertilization capabilities as they traverse the epididymis. *Arrdc4^–/–^* epididymal epithelial cells produce less EVs, particularly those of larger (> 200 nm) size. The decrease in fertility of *Arrdc4^–/–^* sperm appears, at least in part, due to this reduction in EV production as reconstituting *Arrdc4^–/–^* sperm with EVs from wild type epididymal cells restores the fertilizing capabilities of *Arrdc4^–/–^* sperm. These data indicate that Arrdc4 is important for sperm maturation through the epididymis by controlling EV release, and thereby affecting the acquisition of extrinsic signals required for optimal fertilization capacity.

## 2 MATERIALS AND METHODS

### 2.1 Antibodies and reagents

Antibodies were purchased from the following: 5‐Methylcytosine (5‐mC), 5‐Hydroxymethylcytosine (5‐hmC) (Australian Biosearch); α‐tubulin, Androgen receptor, Annexin A1, Cdx2 (Abcam); PKM (ABClonal); GM130, Bip2 (BD Transduction Labs); Alix, CD81, β‐actin (Cell Signaling); Cytokeratin (pan) FITC conjugated (Sigma); Oct‐3/4 (Santa Cruz Biotechnology); Phosphotyrosine clone 4G10, Goat Anti‐Rabbit IgG Antibody (H+L) Alkaline Phosphatase conjugate (Millipore); Lectin PNA from *Arachis hypogaea* (peanut) Alexa Fluor 647 Conjugate, Donkey anti‐Rabbit IgG Alexa Fluor 488, Donkey anti‐Rabbit IgG Alexa Fluor 647, Donkey anti‐Mouse IgG Alexa Fluor Plus 647 (Thermo Scientific); Rabbit IgG HRP Linked Whole Ab Mouse IgG HRP Linked Whole Ab (GE Healthcare).

Fluorescent stains used for flow cytometry were purchased from Thermo Scientific: MitoSOX Red mitochondrial superoxide indicator, SYTOX Green Nucleic Acid Stain, LIVE/DEAD Fixable Far Red Dead Cell Stain Kit, JC‐1 Mitochondrial Membrane Potential Indicator, DAPI (4,6‐diamidino‐2‐phenylindole). PKH67 fluorescent membrane dye was purchased from Sigma.

### 2.2 Animal work

All animal work was performed in the Core Animal Facility at the University of South Australia, with approvals from the institutional animal ethics committee (AEC numbers U23‐16, U06‐19, U25‐19, U41‐19) and the institutional biosafety committee (IBC0090 and IBC0143). The animal facilities were operated according to international animal welfare rules (Federation for Laboratory Animal Science Associations guidelines and recommendations). Mouse colonies were maintained in specific pathogen‐free conditions in individually vented cages with 12–12 h light‐dark cycle. The following mouse strains were used in this study: *Arrdc4^–/–^* mice (Mackenzie et al., 2016); C57BL/6J females and CBA/CaH males were used to generate CBAB6F1 female progeny used in the in vitro fertilization experiments. Male *Arrdc4^–/–^* mice used for experiments were between 4 and 6 months of age, male CBA/CaH males and C57BL/6J females used for breeding were 2–12 months of age, and B6CBAF1 females used for in vitro fertilization studies were 3–7 weeks of age. Average litter size was calculated by counting the number of pups at birth (Table 1).

**TABLE 1:** Litter and pregnancy data from specific Arrdc4 genotype pairings

Male × Female	Litter size	Pregnancy rate (%)	*Arrdc4^–/–^* pup ratio Actual (Expected) %
*Arrdc4^+/–^* × *Arrdc4^+/–^* (n = 95)	6.3 ± 2.5	96.8	20.9 (25)
*Arrdc4^+/–^* × *Arrdc4^–/–^* (n = 13)	5.2 ± 3.1	84.6	30.3* (50)
*Arrdc4^–/–^* × *Arrdc4^+/–^* (n = 33)	4.1 ± 2.7**	81.8	42.7 (50)
*Arrdc4^–/–^* × *Arrdc4^–/–^* (n = 15)	1.6 ± 2.0 ***	53.3***	

Data represented as mean ± S.D. Statistically significant data depicted in bold, *P < 0.05, **P < 0.01, ***P < 0.001 compared with heterozygous pairs. Statistic tests employed: One‐way ANOVA/Tukey's post‐hoc test (litter size), Chi‐squared test (pregnancy rate, Mendelian ratios).

Genotyping was carried out on tail or ear clippings from *Arrdc4^–/‐^* mice using the KAPA HotStart Mouse Genotyping Kit (Sigma) using the genotyping primers listed in Supplemental Table S1, or by the Transnetyx Automated Genotyping Services (Cordova, TN).

### 2.3 Isolation of sperm and epididymal epithelial cells

Cauda epididymides and vas deferens were removed from male mice and placed into dishes containing 500 μl of GIVF Plus media (VitroLife) covered in liquid paraffin. The tissue was cut and sperm drawn out into the media. Sperm was incubated for 70 min at 37°C to capacitate and then used for in vitro fertilization, sperm motility analyses, and immunofluorescence. When required, sperm were incubated in 30 μg EV for the last 15 min of capacitation. After sperm removal, epididymides were rinsed in PBS to remove paraffin and any remaining sperm, then minced in enzyme solution containing 0.25 mg/ml trypsin and 2 mg/ml collagenase type II (Sigma) in PBS and incubated at 37°C on a shaker for 1 h. Tissue clumps were gently dispersed using a 1 ml wide bore pipette tip before being filtered through a 70 μm mesh filter. Filters were washed with 2 ml of PBS and 2 ml EEC media (Iscove's Modified Dulbecco's Medium supplemented with 0.275IU/ml insulin glargine, 200 μM L‐glutamine, 15 mM HEPES, 10 μg/ml transferrin, 10 ng/ml EGF recombinant human protein, 10 nM hydrocortisone, 10 ng/ml 8‐Bromoadenosine 3′,5′‐cyclic monophosphate, 1 nM 5α‐Androstan‐17β‐ol‐3‐one, 10 U/ml penicillin, 1 μg/ml streptomycin, 2.5 μg/ml amphotericin B and 5% FCS) to flush through any remaining cells. The flow‐through was then centrifuged at 1200 rpm for 5 min, then cells were resuspended in EEC media and plated onto gelatin‐coated tissue culture plates. Plates were made the day before cell isolation by adding 2% gelatin in acetic acid to the culture plate and allowing to sit at room temperature overnight. Prior to cell plating, dishes are washed twice in PBS to remove any remaining acetic acid. Once plated, cells were incubated at 34°C, 10% CO_2_ overnight then rinsed in PBS to remove debris and unadhered cells. Fresh EEC media was added and cells were left to grow to confluency. Cells were then split 1–2 times per week until senescent and then maintained in culture until single cell colonies started to form. These were then picked and amplified to produce a spontaneously immortalized cell line of single cell origin. To confirm their origin and nature, cells were tested for expression of cytokeratin (epithelial marker) and androgen receptor (epididymal marker) by immunostaining (Figure S3).

### 2.4 Extracellular vesicle purification

EVs were isolated from cell culture media as previously described (Mackenzie et al., 2016). 2 × 10^6^ EECs were seeded in a 10 cm^2^ dish and allowed to adhere overnight. Cells were then cultured in EEC media containing exosome‐free FBS for 24 h prior to harvest. The media was then collected and centrifuged sequentially at 500 × *g* for 10 min then 2600 × *g* for 15 min at 4°C to remove cell debris and aggregates. Ultracentrifugation of the supernatant was carried out using a TLA100.4 or MLA‐80 rotor in an Optima MAX‐XP (Beckman Coulter) at 100,000 × *g* for 2 h at 4°C to enrich in both larger and smaller EVs in the sample (hereafter referred to as EV^100K^). To enrich the sample in the larger EV subfraction only, supernatants were collected after removing cell debris and aggregates and centrifuged at 10 000 × *g* for 30 min at 4°C (hereafter referred to as EV^10K^). The supernatant was discarded, and the pellets were rinsed with ice‐cold phosphate‐buffered saline (PBS). The pellets were then either resuspended in 1 ml PBS for Nanoparticle Tracking Analysis, 50 μl PBS for addition to isolated sperm or 20 μl SDS lysis buffer (2% SDS, 0.3 M sucrose, 0.1875 M Tris‐HCl, pH 6.7) and sonicated twice for 5 min for immunoblotting. To fluorescently label EVs, pellets were resuspended in 0.5 ml PBS then mixed 1:1 with PKH67 dye solution (2 μl dye in 0.5 ml Diluent C) for 2 min at room temperature with gentle agitation. After incubation, excess dye was quenched by adding 2 ml of 1% BSA/PBS and ultracentrifuged at 1,00,000 × *g* for 2 h at 4°C. The labelled pellets were rinsed in 1x PBS and resuspended in 50 μl PBS.

### 2.5 Assessment of daily sperm production

Daily sperm production (DSP) was determined using a frozen testis from wild type and *Arrdc4^–/–^* males as previously described (Cooke et al., 1991; Joyce et al., 1993; Robb et al., 1978). Briefly, testes were removed and weighed, then placed in liquid nitrogen, and subsequently kept at −80°C until examination. Testes were homogenized for 1 min in 1 ml of physiological saline containing 0.05% (v/v) Triton X‐100, using a 1.5 ml sterile pestle (Ayxgen #PES‐15‐B‐SI) in a 1.5 ml Eppendorf tube. Samples were diluted to 25 ml in the same buffer. Step 14–16 spermatids (stage II–VIII) survive this homogenization, and their nuclei can then be counted. To count the spermatids, the homogenate was diluted in 4% Trypan blue and counted using a haemocytometer to determine the average number of spermatids per gram of testis. Developing spermatids spend 4.84 days in steps 14–16 during spermatogenesis in the mouse. Thus, the spermatids per gram of testis was divided by 4.84 to obtain the DSP.

### 2.6 Capacitation‐associated tyrosine phosphorylation

To assess the ability of sperm to undergo tyrosine phosphorylation during capacitation, sperm were taken after 70 min incubation in GIVF plus media (containing 10 mg/ml HSA), washed in 1x PBS followed by centrifugation at 250 *g* and resuspended in SDS buffer consisting of 2% SDS, 0.19 M Tris‐HCl pH 6.7, 10% sucrose, 1x Halt Protease and Phosphatase Inhibitor Cocktail (Thermo Scientific). Samples were sonicated using a Bioruptor ultrasonicator (Diagenode) with high power mode for 10 cycles (sonication cycle: 30 s ON, 30 s OFF), then boiled for 5 min to extract proteins. Samples were then clarified by centrifugation at maximum speed for 5 min at 4°C. Protein was quantified using a Pierce BCA Protein Assay (Thermo Scientific), and 5 μg added to Laemmli buffer and boiled for a further 5 min to denature proteins. Proteins were resolved on 10% Mini‐PROTEAN TGX precast gels (Bio‐Rad) and transferred to PVDF membranes using the Trans‐Blot Turbo Transfer System (Bio‐Rad). Blots were then blocked overnight at 4°C in 5% cold water fish skin gelatin in TBST (Goodson et al, 2012). Tyrosine phosphorylation was detected using the 4G10 antibody diluted 1:1000 in 5% fish skin gelatin/TBST for 1 h at room temperature, followed by HRP‐conjugated goat anti‐mouse IgG diluted 1:10,000 in 5% fish skin gelatin/TBST for 1 h at room temperature. Chemiluminescence signal was detected using the SuperSignal West Femto Maximum Sensitivity Substrate (Thermo Scientific) on the ChemiDoc MP Gel Imaging System (Bio‐Rad Laboratories).

### 2.7 Acrosome reaction

To assess acrosome integrity, 5 μl of sperm suspension was taken immediately after harvesting, washed briefly in GIVF Plus media and dropped onto a glass slide to form the “basal” samples. To trigger the acrosome reaction, 5 μl of sperm suspension was resuspended in 500 μl GIVF Plus media containing 20 μM calcium ionophore A23187 with or without EVs and incubated at 37°C for 1 h. Sperm was then centrifuged at 500 x *g* for 3 min to remove supernatant and then dropped onto a glass slide to form the “A23187” samples. Slides were then stored at 4°C until processing.

To visualize the acrosome reacted sperm, slides were briefly rinsed in water then fixed in 10% neutral buffered formalin for 10 min at room temperature. Slides were then washed three times for 5 min in PBS/PVP (1 mg/ml Polyvinylpyrrolidone in PBS). Slides were then stained with lectin‐PNA‐647 in PBP/PVP (1:1000) for 15 min at room temperature in the dark. Slides were then washed for a further 3 × 5 min in PBS/PVP and mounted in Prolong Gold Antifade Mountant with DAPI (Thermo Scientific) before imaging with a Leica Biosystems SP8 TCS confocal microscope.

### 2.8 Sperm motility analysis

To analyse sperm for motility parameters, 1 μl of sperm suspension isolated from mouse epididymides was resuspended in 50 μl of GIVF plus media and dropped onto poly‐HEMA coated glass slides. Movies of sperm movement were taken using a DP70 camera (Olympus) mounted on a CX41 inverted microscope (Olympus). Movies were then analysed using the Computer Assisted Sperm Analyser (CASA) plugin for ImageJ (Wilson‐Leedy & Ingermann, 2007) using the parameters listed in Supplemental Table S2 which were based on previous studies on rodent sperm motility (Chapin et al., 1992; Goodson et al., 2011; Vadnais et al., 2014).

### 2.9 Flow cytometry

To assess sperm mitochondrial activity, we examined sperm intracellular levels of Superoxide and mitochondrial membrane potential, using flow cytometry based on previously published methods (Koppers et al., 2008, 2011). Briefly, 1 × 10^6^ sperm cells per assay per male were first suspended in Biggers, Whitter and Whittingham (BWW) media at 37 degrees Celsius. Then, the samples were incubated either with Mitosox Red (MSR, Invitrogen) to measure intracellular superoxide levels (Koppers et al., 2008, 2011), or with JC‐1 (Thermo Fisher) to measure mitochondrial membrane potential (Koppers et al., 2011). Dead sperm cells were excluded using either Sytox Green (MSR assay, Thermo Fisher), or Live/Dead Fixable Far Red Dead Cell Stain (JC1 assay, Invitrogen) to assess cell viability. Flow cytometry was performed using a FACSCanto II (Becton, Dickinson and Company). Emission measurements were made using 530/30 band‐pass (green/FITC), 585/42 band‐pass (red/PE) and > 670 long‐pass (far red/APC). Forward scatter and side scatter measurements were taken to generate a scatter plot, used to gate for the sperm cell population, excluding any larger contaminating cells. All data were acquired using BD FACS Diva software (BD Biosciences) with a total of 10,000 events collected per sample. All data were analyzed using FlowJo LLC (BD Bioscience).

### 2.10 Zona pellucida binding assay

To assess the zona pellucida binding capacity of sperm, oocytes with intact zona pellucida were rinsed several times by mouth pipetting to remove cumulus cells, then incubated with capacitated sperm for 4 h. Oocytes (with any bound sperm) were then fixed in 4% paraformaldehyde overnight at 4°C. Oocytes were then washed twice for 5 min in PBS/PVP before being mounted in Prolong Gold with DAPI to visualize sperm nuclei.

### 2.11 In vitro fertilization

To harvest oocytes, B6CBAF1 females were injected firstly with Folligon followed by a second injection 48 h later of Chorugon, and then 16 h after the second injection, mice were humanely killed and ovaries and oviducts dissected out and placed into a dish containing 200 μl GIVF Plus media covered in liquid paraffin. Under a dissecting microscope, the oviduct ampulla was located and pierced with a needle. A droplet of liquid containing the cumulus‐oocyte‐complexes (COCs) was dragged from the ampulla into the drop of media. COCs were then incubated at 37°C for 60 min to acclimatize. Sperm harvested and capacitated as described were added to the COC‐containing media at a rate of 1000 sperm per oocyte and incubated for a further 3 h to allow fertilization to occur. After 3 h oocytes were washed three times in GIVF Plus media by transferring between droplets covered in liquid paraffin using a mouth pipette and glass capillary tubes. Putative zygotes were then incubated for 24 h at 37°C, 5% CO_2_ to allow for cleavage and the number of 2‐cell stage embryos was counted.

### 2.12 Time‐lapse imaging of embryo development and morphokinetics analysis

CBAF1 females were injected with Pregnant Mare Serum Gonadotropin (PMSG; Lyppard Australia), followed by injection with Human Chorionic Gonadotropin (HCG; Lyppard Australia) to induce superovulation and maxime the collection of mature COCs. Briefly, COCs were collected at 15–16 h post‐HCG injection and inseminated via IVF. After 4 h, the presumed zygotes were mechanically cleaned of any sperm attached to the zona pellucida using a mouth pipette. For each male, eight presumed zygotes that presented at least one polar body (indication of MII oocyte) were selected for time‐lapse imaging of embryo preimplantation development using the Primovision (Vitrolife system).The presumed zygotes were cultured in COOK Research Cleave medium at 37°C, 5% CO_2_ and 6% O_2_ for 96–100 h, with one image taken every 10 min. Data were collected using Primovision Analyzer Software, tagging time points in the manner according to Milewski and Ajduk (2017).

### 2.13 Histology

Tissues were fixed in either Bouin's fixative (testis) or 4% paraformaldehyde (epididymides) overnight before transferring into 70% ethanol. Tissue samples were processed, embedded in paraffin and 5 μm sections were cut and stained for Haematoxylin and Eosin by the Histology Department, University of Adelaide. Digital images were acquired using a Pannoramic Confocal Scanner with CaseViewer software (3DHISTECH, Hungary).

### 2.14 Immunostaining

For immunostaining of tissue samples to identify the *Arrdc4* gene expression pattern, we utilized the presence of a β‐galactosidase reporter in the knockout animals, due to a lack of Arrdc4 antibody. Tissues were fixed in Histochoice MB fixative (Sigma) for a minimum of 24 h, cryopreserved in 30% sucrose in PBS for a minimum of 24 h then frozen in OCT compound. 10 μm sections were then cut onto Polysine™ Microscope Adhesion Slides (Thermo Fisher Scientific) with a Leica Biosystems CM1850 cryostat and stored at ‐80°C until use. Slides were washed 3 × 5 min in PBS to remove OCT and sections blocked in 5% goat serum in PBST for 2 h at room temperature. Slides were then incubated in primary antibody (β‐galactosidase 1:2000 in 5% goat serum in PBST) overnight in a humidified chamber at 4°C. Slides were then washed 3 × 5 min in PBS followed by incubation in secondary antibody (goat anti‐rabbit AlexaFluor‐488 1:500) for 2 h at room temperature in the dark. Slides were washed for a final 3 × 5 min in PBS, then mounted in Prolong Gold with DAPI.

For immunostaining of EECs, cells were seeded onto glass coverslips at a density of 5 × 10^4^ cells/slip and incubated overnight to adhere. Media was removed and cells briefly washed in PBS before fixation in methanol:acetone (1:1) for 15 min at room temperature. Cells were then blocked in 1% donkey serum in PBS for 45 min and then incubated in primary antibody (Cytokeratin‐FITC 1:50 and Androgen Receptor 1:500, in 1% donkey serum) overnight at 4°C. The cells were washed three times in PBS, then incubated in secondary antibody (donkey anti‐rabbit 568 1:500 in 1% donkey serum) for 2 h at room temperature. Cells were then washed again three times in PBS and then mounted in Prolong Gold with DAPI and imaged using a Leica Biosystems SP8 TCS confocal microscope.

To visualize two pronuclei, fertilized oocytes were collected 8–10 h after insemination and the zona pellucida removed using acid tyrode's solution (136 mM NaCl, 2.7 mM KCl, 1.6 mM CaCl_2_.2H_2_O, 0.5 mM MgCl_2_.6H_2_O, 5.5 mM glucose, 0.4% PVP). Oocytes were then washed in PBS/PVP and then fixed in 3.7% paraformaldehyde in PBS for 20 min at room temperature. Oocytes were washed twice more in PBS/PVP and then permeabilized in 0.2% triton X‐100 in PBS for 10 min. Following three more washes in PBS/PVP, oocytes were incubated in 4N HCl solution for 10 min at room temperature, followed by four washes (3 min each) in 0.05% Tween‐20 in PBS (PBST). Oocytes were then blocked overnight in blocking solution (1% BSA, 0.02% Triton X‐100 in PBS) at 4°C. The pronuclei were then stained with 5‐mc and 5‐hmc antibodies (5‐mc 1:200, 5‐hmc 1:600 in blocking solution) overnight at 4°C. Oocytes were washed three times for 10 min with PBST and incubated with secondary antibodies (goat anti‐mouse AlexaFluor 647 and goat anti‐rabbit AlexaFluor 488, both 1:500 in blocking solution) for 1 h at room temperature. After three final washes in PBST (10 min each), oocytes were mounted in Prolong Gold with DAPI and imaged.

To assess blastocyst cell composition, blastocysts were collected 96 h after insemination and fixed in 4% paraformaldehyde for 1 h, then stored in PBS/PVP overnight at 4°C. Blastocysts were then incubated in 0.1 M glycine in PBS/PVP for 5 min at room temperature, followed by three washes in PBS/PVP. Blastocysts were permeabilized in 0.5% Triton X‐100 in PBS/PVP for 30 min, washed three times in PBS/PVP, then blocked for 1 h in 10% donkey serum in PBS/PVP. To differentiate between cell types, blastocysts were then stained with Oct4 (1:500 in 10% donkey serum, inner cell mass) and Cdx2 (1:500 in 10% donkey serum, trophoblast) overnight at 4°C. After overnight incubation, blastocysts were washed three times and incubated with secondary antibodies (donkey anti‐goat AlexaFluor 488 and donkey anti‐rabbit AlexaFluor 647, both 1:500 in 10% donkey serum) for 1 h at room temperature. Blastocysts were then washed in PBS/PVP and mounted in Prolong Gold with DAPI before imaging.

### 2.15 Mass spectrometry of sperm

Protein samples were resuspended in 6 M Urea, 100 mM DTT and 100 mM Tris‐HCl pH7.0 and subjected to protein digestion using FASP (filter aided sample preparation). Peptides were collected and acidified with formic acid (FA) to a 1% final concentration. Solvent was removed in a CentriVap concentrator (Labconco) and peptides were resuspended in MilliQ water containing 2% acetonitrile (ACN) and 1% formic acid. An aliquot of peptides from pooled samples was also used to generate a fraction library. The pooled sample was subjected to stepwise high‐pH fractionation. Peptides were resuspended in 10 mM Ammonium Formate pH10 and loaded onto a stage‐tip (home‐packed, 4 C18‐matrix discs; activated using Methanol, washed in 5 mM Ammonium Formate/50% acetonitrile (ACN) and equilibrated two times with 10 mM Ammonium Formate pH 10). The samples were fractionated by stepwise elution in 10 mM Ammonium Formate with increasing concentrations of ACN: 5%, 7.5%, 10%, 12.5%, 15%, 17.5%, 20%, 60%. The fractionated peptide solutions were then concentrated by centrifugal lyophilization. Peptides were injected and separated by reversed‐phase liquid chromatography on a M‐class UPLC system (Waters, USA) using a 250 mm × 75 μm column (1.6μm C18, packed emitter tip; IonOpticks, Australia) with a linear 90‐min gradient at a flow rate of 400 nl/min from 98% solvent A (0.1% Formic acid in Milli‐Q water) to 35% solvent B (0.1% Formic acid, 99.9% acetonitrile). The UPLC was coupled on‐line to a Q‐Exactive mass spectrometer (Thermo, Bremen, Germany). The Q‐Exactive was operated in a data‐dependent mode, switching automatically between one full‐scan and subsequent MS/MS scans of the ten most abundant peaks. The instrument was controlled using Exactive series version 2.6 and Xcalibur 3.0. Full‐scans (m/z 350–1850) were acquired with a resolution of 70,000 at 200 m/z. The ten most intense ions were sequentially isolated with a target value of 10,000 ions and an isolation width of 3 m/z and fragmented using HCD with normalized collision energy of 27 and stepped collision energy of 15%. Maximum ion accumulation times were set to 50 ms for full MS scan and 150 ms for MS/MS. Underfill ratio was set to 2% and dynamic exclusion was enabled and set to 30 s.

The raw files were analysed using the MaxQuant software version 1.5.8.3 (Cox & Mann, 2008; Cox et al., 2011), The database search was performed using mouse protein sequences obtained from Uniprot including isoforms with strict trypsin specificity allowing up to two missed cleavages. The minimum required peptide length was set to seven amino acids. Carbamidomethylation of cysteine was set as a fixed modification while N‐acetylation of proteins N‐termini and oxidation of methionine were set as variable modifications. During the MaxQuant main search, precursor ion mass error tolerance was set to 4.5 ppm and fragment ions were allowed a mass deviation of 20 ppm. PSM and protein identifications were filtered using a target‐decoy approach at a false discovery rate (FDR) of 1%.

Further analysis was performed using a custom pipeline developed in R, which utilizes the LFQ intensity values in the MaxQuant output file proteinGroups.txt. Proteins not found in at least 50% of the replicates in one group were removed. Missing values were imputed using a random normal distribution of values with the mean set at mean of the real distribution of values minus 1.8 SD, and a SD of 0.3 times the SD of the distribution of the measured intensities. The probability of differential protein expression between groups was calculated using the Limma R package. Probability values were corrected for multiple testing using Benjamini–Hochberg method.

### 2.16 Mass spectrometry of EVs

Equal amount (30 μg) of protein samples were separated in SDS‐PAGE. The separated protein bands were extracted using scalpel blade in to five gel bands after visualizing using Coomassie Brilliant Blue stain. The bands were then subjected to reduction, alkylation, and trypsinization as previously described (Fonseka et al., 2019). Briefly, 10 mM DTT (Bio‐Red) was used for reduction, 25 mM iodoacetamide (Sigma) was used for alkylation and the samples were trypsinized using 150 ng of trypsin (Promega). The tryptic peptides were extracted using 50% (w/v) acetonitrile and 0.1% (v/v) trifluroacetic acid.

LC‐MS/MS was carried out using the Fusion Lumos Orbitrap mass spectrometers (Thermo Fisher, USA). The LC system was equipped with an Acclaim Pepmap nano‐trap column (Dinoex‐C18, 100 Å, 75 μm x 2 cm) and an Acclaim Pepmap RSLC analytical column (Dinoex‐C18, 100 Å, 75 μm x 50 cm). The tryptic peptides were injected into the enrichment column at an isocratic flow of 5 μL/min of 2% v/v acetonitrile containing 0.1% v/v formic acid for 6 min, applied before the enrichment column was switched in‐line with the analytical column. The eluents were 0.1% v/v formic acid (solvent A) in water and 100% v/v acetonitrile in 0.1% v/v formic acid (solvent B). The flow gradient was (i) 0–6 min at 3% B; (ii) 6–35 min, 3–22% B; (iii) 35–40 min, 22–40% B; (iv) 45–50 min, 40–80% B; (v) 50–55 min, 80‐80% B; (vi) 55–56 min 85‐3% and equilibrated at 3% B for 10 min before injecting the next sample. The Fusion Lumos mass spectrometer was operated at positive‐ionization mode, with the spray voltage set at 1.9 kV and the ion transfer capillary temperature at 275°C. The mass spectrometer was operated in the data‐dependent acquisition mode, whereby full MS1 spectra were acquired in a positive mode at 120,000 resolution at m/z 200, with an AGC target of 5e^5^. The “top speed” acquisition mode (cycle time: 3 s) on the most intense precursor ion was used, whereby ions with charge states of 2 to 5 were isolated using an isolation window of 1.2 m/z and fragmented with using HCD with a stepped collision energy of 30 ± 5%. Fragment ion spectra were acquired in Orbitrap at 15,000 resolution at m/z 200. Dynamic exclusion was 30 s.

Raw files were analysed using MaxQuant platform (version 1.6.17.0; Cox & Mann, 2008) and searched against UniProt database using default LFQ search parameters with the following parameters: LFQ min. ratio count = 2 with unique + razor peptides used for quantitation. Match between runs feature is activated. For this search Trypsin/P cleavage specificity (cleaves after lysine or arginine, even when proline is present) was used with a maximum of two missed cleavages. Oxidation of Methionine and Acetylation of protein N‐Term were specified as variable modifications. Carbamidomethylation of cysteine was set as a fixed modification. False discovery rates (FDR) were determined through the target‐decoy approach set to 1% for both peptides and proteins. LFQ intensities imported from the proteinGroups.txt output were Log2 transformed.

### 2.17 Quantitative PCR

Total RNA was extracted from cells or tissues using TRIzol reagent (Thermo Scientific) according to manufacturer's instructions. First strand cDNA synthesis was performed using the High Capacity cDNA Reverse Transcription kit (Thermo Scientific) according to manufacturer's instructions. Quantitative PCR (qPCR) was performed using the KAPA SYBR FAST Universal Kit (Sigma) in Rotor Disc 100 discs on a Rotor‐Gene Q Real‐time PCR cycler (QIAGEN) according to the following cycle program: 95°C for 3 min, then 40 cycles of 95°C for 3 s and 60°C for 25 s. Results were analysed using the Rotor‐Gene Q Series software (QIAGEN).

### 2.18 Transmission electron microscopy

Sperm were harvested as described and fixed overnight in fixative solution containing 1.25% v/v glutaraldehyde, 4% w/v sucrose, 4% w/v paraformaldehyde in phosphate‐buffered saline (PBS) at room temperature (RT). Specimens were then washed in PBS containing 4% w/v sucrose (2x) for 5 min and postfixed in 1% osmium tetroxide for 1 h. Specimens were washed as before, and then dehydrated in an graded ethanol series from 70% to absolute ethanol (3 × 15 mins each), followed by 1:1 100% v/v ethanol/propylene oxide (30 min) and 100% propylene oxide (1 h). Samples were then pre‐embedded in 1:1, 100% propylene oxide/epon‐araldyte resin (1 h, Proscitech) before embedding in epon‐araldyte resin (3 × 8 h). Specimens were then placed in the appropriate moulds with new resin and placed in the oven to cure at 70°C overnight.

Thick survey sections (1 μm) and thin TEM (70 nm) sections were cut using a Leica Biosystems UC6 ultramicrotome. Thin sections were placed on copper grids (200 mesh, Proscitech) and stained with uranyl acetate (4%) followed by Reynolds lead citrate for 10 min each. Sections were viewed using a FEI Tecnai G2 Spirit TEM operating at 100 KV.

EV^100K^ were harvested as described and resuspended in PBS. 5 μl of sample was spotted onto cleaned (GATAN Solarus 950 Advanced Plasma Cleaner) formvar/carbon 200 mesh grids (Proscitech) and left to adhere for 10 min. The grids were then washed twice in PBS and fixed in 2.5% glutaraldehyde in PBS for 5 min. Grids were washed twice in purified water and stained with 2% uranyl acetate (1 min) before being examined using a FEI Tecnai G2 Spirit TEM operating at 100 KV.

### 2.19 Statistical analysis

Statistical analyses were all performed using GraphPad Prism or SPSS software. One‐way ANOVA with a Tukey's post‐hoc test or Dunnett's Multiple Comparison Test were used for analysis of litter sizes, sperm motility parameters, zona binding assay, and blastocyst analysis parameters. Two‐way ANOVA with Sidak's multiple comparisons test was used to analyse acrosome reaction. Chi‐squared tests were used for analysis of Mendelian ratios and pregnancy rates. Unpaired t‐tests were used to compare wild type and *Arrdc4^–/‐^* data, and paired t‐tests were used to compare *Arrdc4^–/–^* and *Arrdc4^–/–^* + EV pairs. Linear mixed‐effects model of repeated measures with post‐hoc multiple comparisons test was used to compare blastocyst development stages (Figure S6G).

## 3 RESULTS

### 3.1 Male *Arrdc4^–/–^* mice have reduced fecundity

**FIGURE 1:**
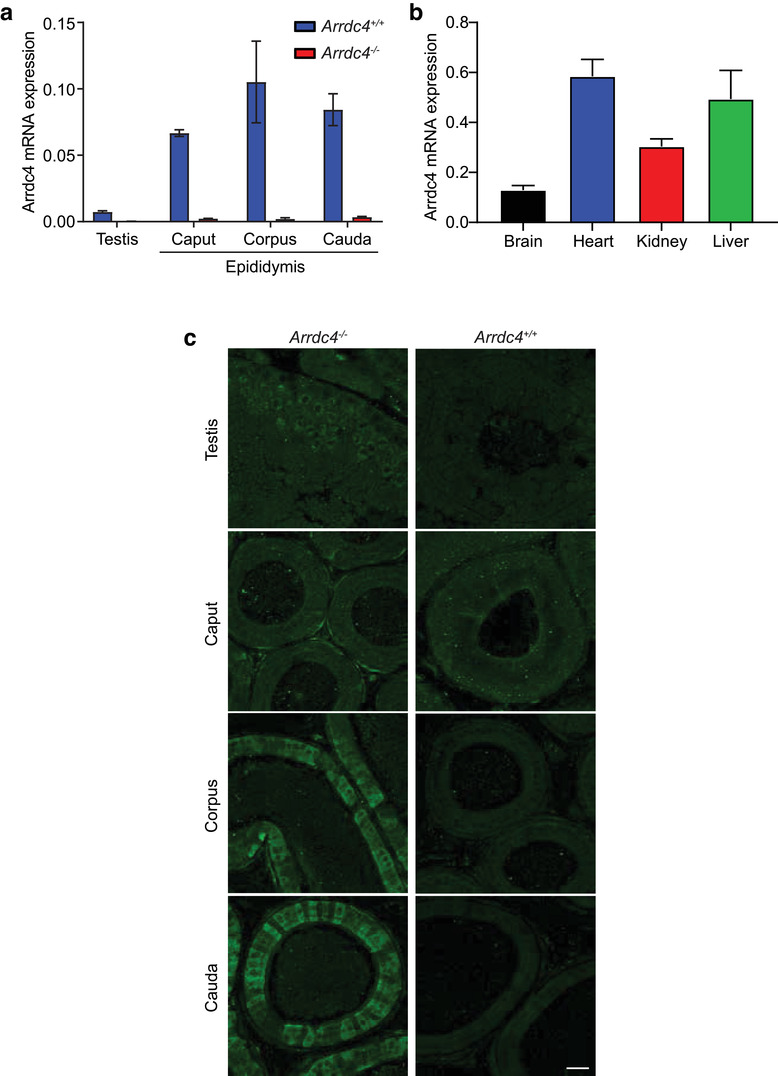
Arrdc4 expression in the epididymis. (a) Expression of *Arrdc4* transcript in male reproductive tissues of *Arrdc4^+/+^* and *Arrdc4^–/–^* mice showing *Arrdc4* knockdown in the *Arrdc4^–/–^* tissues. TBP is used as the reference gene. (b) Expression of *Arrdc4* transcript in brain, heart, kidney and liver of *Arrdc4^+/+^* mice. TBP is used as the reference gene. (c) Protein expression of Arrdc4 using immunostaining of the ß‐galactosidase reporter in the testis and epididymis of *Arrdc4^–/–^* mice. ß‐galactosidase expression as a marker of Arrdc4 is limited to the smooth muscle cells in the caput epididymis, with increasing expression in the principal epithelial cells of the corpus and cauda epididymis. *Arrdc4^+/+^* tissue does not express the ß‐galactosidase reporter and so acts as a negative control. Scale bar, 50 μm

To investigate the role of Arrdc4 in EV production and cargo trafficking, we generated *Arrdc4* gene deleted mice (Mackenzie et al., 2016). We observed that breeding pairs containing *Arrdc4^–/–^* males and females often resulted in either no offspring or a reduced number of pups per litter compared to breeding pairs containing *Arrdc4^+/–^* (Table 1). This seemed to be predominantly driven by deficiencies in the males, as breeding pairs containing *Arrdc4^–/–^* males with *Arrdc4^+/–^* females also had significantly reduced litter sizes, whereas pairs containing *Arrdc4^–/–^* females with *Arrdc4^+/–^* males did not (Table 1). Thus, we further investigated male reproductive fitness in *Arrdc4* ^–/‐^ animals. Loss of Arrdc4 in male reproductive tissues in *Arrdc4^–/–^* mice was confirmed by qPCR (Figure 1a). *Arrdc4* mRNA expression was low in the testis and higher in the epididymis, with consistent expression across all regions of the epididymis. *Arrdc4* mRNA is also ubiquitously expressed across many other organs (Figure 1b). Using β‐galactosidase expressed in knockout mice under the control of the *Arrdc4* promoter, the reporter expression was found to be limited to the smooth muscle cells of the caput epididymis, with increasing expression in the epithelial cells of the corpus and cauda epididymis (Figure 1c). Both male and female *Arrdc4^–/–^* mice showed no other observable phenotypes under standard conditions, with normal growth and survival rates (Figure S1).

### 3.2 *Arrdc4^–/–^* mice have normal spermatogenesis but altered sperm proteome

**FIGURE 2:**
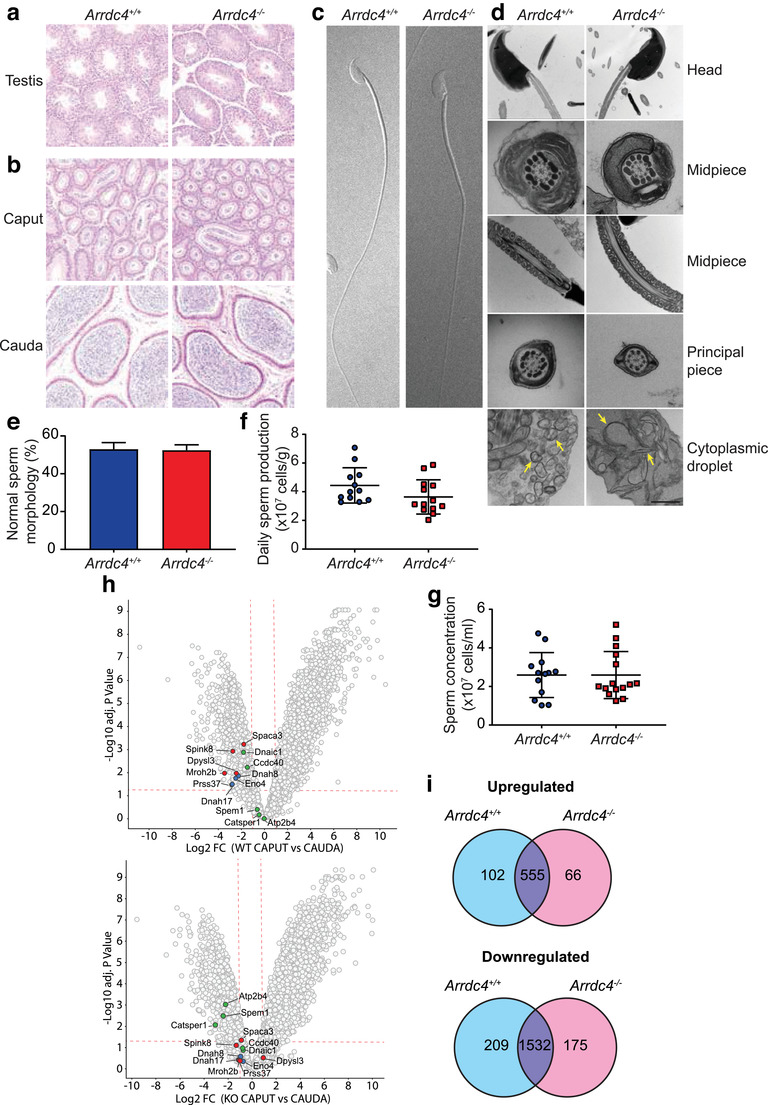
Spermatogenesis is not impaired in *Arrdc4^–/–^* mice but the sperm proteome is altered. (a, b) Haematoxylin and eosin stained sections of (a) testis and (b) caput and cauda regions of the epididymis show no morphological differences between *Arrdc4^+/+^* and *Arrdc4^–/–^* mice. Scale bar, 50 μm. (c, d) Microscopic images of *Arrdc4^+/+^* and *Arrdc4^–/–^* sperm. (c) Differential interference contrast. (d) Transmission electron micrographs. Potential thinning of the acrosomal cap can be observed in *Arrdc4^–/–^* sperm (red arrows) and differences can be seen in the contents of the cytoplasmic droplet between wild type and *Arrdc4^–/–^* sperm (yellow arrows). Scale bars, 500 nm. (e) The number of sperm showing normal morphology under brightfield confocal microscopy was unchanged between *Arrdc4^+/+^* and *Arrdc4^–/–^* mice. 200 sperm were counted per animal, and three animals were used per genotype (mean ± SD). (f) Daily sperm production is unaffected in *Arrdc4^–/–^* mice (n = 12–13, mean ± SD). (g) Sperm concentration from the cauda epididymis is unaffected in *Arrdc4^–/–^* mice (n = 13–15, mean ± SD). (h, i) Differential protein expression assessed using mass spectrometry in the caput and cauda epididymis of *Arrdc4^+/+^* and *Arrdc4^–/–^* mice. (H) Volcano plots demonstrating proteins significantly changed by greater than two‐fold and a *P* value < 0.05 (dotted line) in cauda sperm compared with caput sperm. Several proteins associated with sperm function have been highlighted (“Acrosome vesicle” (GO:0001669; red), “Cilium movement” (GO:0003341; blue) and “Sperm motility” (GO:0097722; green)). (i) Venn diagram depicting the total number of proteins that are up‐ or down‐ regulated in the cauda sperm compared with caput sperm in *Arrdc4^+/+^* and *Arrdc4^–/–^* mice

To investigate the cause of the reduction in male fecundity, sperm morphology, and development was comprehensively examined according to the established tests recommended by the World Health Organisation, including assessing microscopic appearance, sperm motility, sperm number, sperm vitality (if the percentage of motile cells is low), and sperm morphology (World Health Organization, 2010). Haematoxylin and eosin stained sections of testis showed normal progression of spermatogenesis (Figure 2a). The epididymis also showed comparable morphology between wild type and *Arrdc4^–/–^* mice, with no signs of infection, inflammation or scarring (commonly seen in epididymitis patients), and tubules containing ample sperm deposits in both proximal (caput) and distal (cauda) regions, demonstrating that no blockage is occurring (Figure 2b). Sperm ultrastructure, using both differential interference contrast (DIC) microscopy and transmission electron microscopy (TEM) of sperm isolated from the cauda epididymis showed no structural defects, with normal nuclear condensation, head shape, distribution of mitochondria, and arrangement of the axoneme (Figure 2c, d). The acrosomal cap was present in both wild type and *Arrdc4^–/–^* sperm, however, a thinning of the acrosomal cap could be observed in *Arrdc4^–/–^* sperm (Figure 2d, red arrow). The cytoplasmic droplet mostly consists of spherical double‐membrane structures in the wild type sperm as has been described in bovine sperm (Garbers et al, 1970), yet in *Arrdc4^–/–^* sperm these structures were flattened or incomplete (yellow arrows). There was no difference in the percentage of sperm displaying normal morphology (Figure 2e). Daily sperm production in the testis as measured by counting step 14–16 spermatids in homogenized samples was comparable between wild type and *Arrdc4^–/–^* mice (Figure 2f), as was the concentration of sperm isolated from the cauda epididymis (Figure 2g).

Since sperm morphology and development were mostly unaffected in these mice, proteomic analysis was performed on caput and cauda sperm from wild type and *Arrdc4^–/–^* mice to determine whether there were any changes in the sperm proteome that may explain the loss of male fecundity. This comparison revealed a surprisingly small number of proteins that varied between the two genotypes (Figure S2, Supplemental Table S3), which are broadly involved in energy production and other metabolic processes (Coq3, Mipep, Nnt, Pkm, Tha1), or in protein transport and regulation (Ank3, Ap2a2, Cdc42bpb, Eif3l, Gzmn, Pdia5, Plg). To further interrogate any sperm proteome shifts, we compared caput versus caudal sperm in each genotype. There were a number of proteins that were either up or down regulated as the sperm traverse the epididymis from caput to cauda in both the wild type mice, as previously demonstrated (Skerget et al., 2015), and similar changes were observed in *Arrdc4^–/–^* sperm (Figure 2h, Supplemental Tables S4‐S7). Although the vast majority were similarly regulated in wild type and *Arrdc4^–/–^* sperm, comparison of the proteins upregulated or downregulated during transit demonstrated a number of differences (Figure 2i). While no specific pathway was found to be significantly altered using the FunRich Functional Enrichment Analysis Tool (Pathan et al, 2015; Pathan et al., 2017), many of the affected proteins were linked to GO terms associated with sperm function. Figure 2h shows some examples of affected proteins associated with the GO terms “Acrosome vesicle” (GO:0001669; red), “Cilium movement” (GO:0003341; blue) and “Sperm motility” (GO:0097722; green). Together this indicates that while *Arrdc4^–/–^* sperm appear mostly normal morphologically, there are molecular differences that may be involved in the reduction in sperm function.

### 3.3 Arrdc4 is required for EV production by epididymal cells

**FIGURE 3:**
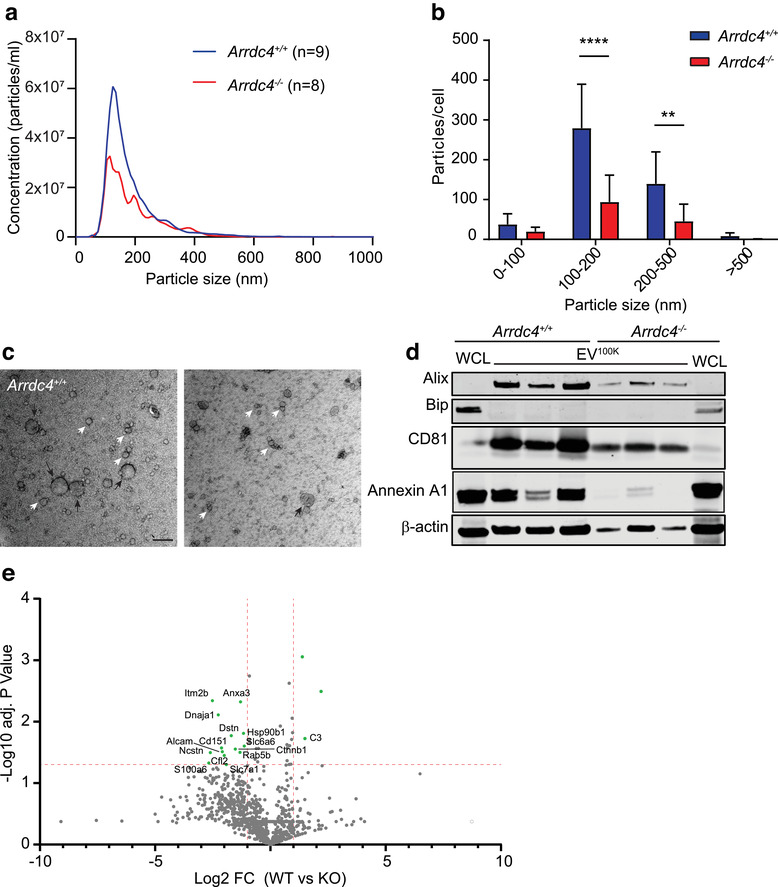
Arrdc4 is required for EV production by epididymal cells. (a) Average size distribution of EV^100K^ harvested from epididymal cells as measured by nanoparticle tracking analysis (NTA). (b) *Arrdc4^–/–^* cells release fewer of the larger EVs (> 100 nm) per cell as measured by NTA. n = 4–6, mean ± SEM, * *P* < 0.05, **** *P* < 0.0001. (c) TEM confirms the reduction in particle size produced by *Arrdc4^–/–^* cells, with a loss of the larger sized EV in the EV^100K^ samples (> 100 nm, black arrows), while the small EVs are still present (< 100 nm, white arrows). Scale bar, 200 nm. (d) Immunoblotting of EV^100K^ harvested from different EEC clones showing the presence of EV markers Annexin A1, Alix and CD81, and the absence of the non‐EV component Bip. EV^100K^ were harvested from 2 × 10^6^ cells per clone and the whole pellet loaded onto gel. *Arrdc4^–/–^* EV^100K^ showed a reduction in levels of all EV markers, confirming a reduction in EV production. (e) Volcano plots demonstrating proteins significantly changed by greater than two‐fold and a *P* value < 0.05 (dotted line) in *Arrdc4^+/+^* EV^100K^ compared with *Arrdc4^–/–^* EV^100K^. Significantly changed proteins are highlighted in green

As sperm traverse the epididymis, it is hypothesized that they obtain essential proteins required for motility and fertility via the uptake of EVs secreted by the epithelial cells bordering the intraluminal compartment (Girouard et al., 2011; Simon et al., 2018; Sullivan et al., 2005). As our previous work established a requirement for Arrdc4 in EV production in other cell types (Mackenzie et al., 2016), we hypothesized that sperm from *Arrdc4^–/–^* mice are unable to fully mature due to reduced EV production in the epididymis. To test this, we first isolated and cultured caput epididymal epithelial cells (EECs) from both wild type and *Arrdc4^–/–^* mice. The origin and nature of these cells was confirmed by immunostaining using cytokeratin as a marker for epithelial cells and androgen receptor as a marker for epididymal cells (Figure S3). EV^100K^ were harvested from the supernatant using ultracentrifugation and counted by Nanoparticle Tracking Analysis. *Arrdc4^–/–^* EECs showed a significant reduction in the production of larger sized particles (100–500 nm), but no change in the smaller (< 100 nm) particles (Figure 3a, b). This was confirmed by TEM, which also showed a reduction in the presence of larger (> 200 nm) vesicles by *Arrdc4^–/–^* EECs (Figure 3c, black arrows), while the smaller vesicles appeared unaffected (Figure 3c, white arrows). As the large EV subpopulation are considered to be formed primarily by direct budding of the plasma membrane, it is possible direct ultracentrifugation of the supernatant following the 2500 × *g* centrifugation step could result in the loss of larger EVs due to rupture of the membrane. However, the presence of larger sized EVs in our Nanosight and TEM analyses suggests that the process of collecting EV samples used here does not deplete all the large EVs from our fractions, but there may be an underestimation of total numbers of larger EVs produced by the cells. Consistent with this, immunoblotting of EV^100K^ harvested from different EEC clones show the presence of known EV markers Annexin A1, Alix and CD81 (Figure 3d). The absence of Bip indicates that there is no cellular contamination in the fractions (however, it is possible that other extracellular material may be present due to the nature of collection). Figure 3d further confirms the reduction in EV production by *Arrdc4^–/–^* EECs, as EV^100K^ from the same number of cells (2 × 10^6^) were loaded into each lane, and the reduction of protein levels in the *Arrdc4^–/–^* EECs is clearly evident.

To characterize the protein content of EV^100K^ from wild type and *Arrdc4^–/–^* EECs in more detail, we performed mass spectrometry (Figure 3e). When equal amounts of protein are compared, there are only a small number of proteins that are significantly different, and of these only Dnaja1 has a known Gene Ontology associated with sperm function. This further indicates that a reduction in EV number rather than changes to EV content were responsible for the observed phenotype.

### 3.4 Fertility defects in *Arrdc4^–/–^* sperm are due to reduced EV production

**FIGURE 4:**
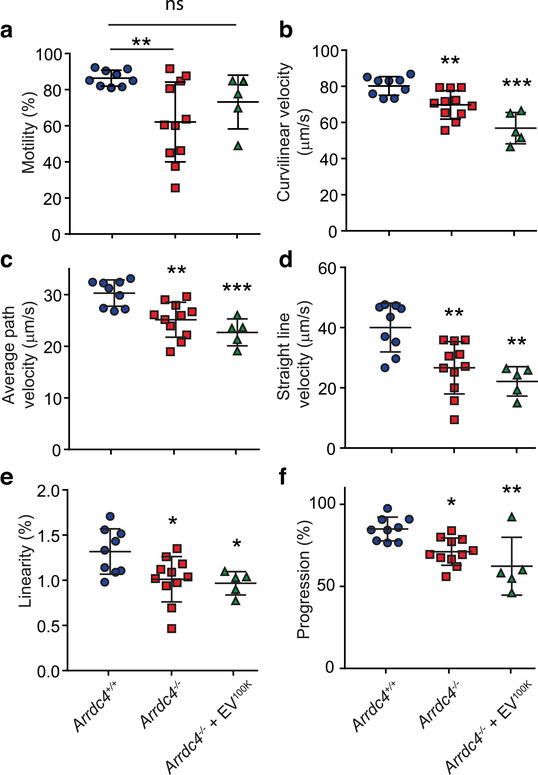
Sperm from *Arrdc4^–/–^* mice show motility defects which can be partially rescued by wild type EVs. (a–f) Using the computer‐assisted sperm analysis plugin with ImageJ, *Arrdc4^–/–^* sperm showed a reduction in (a) total motility, (b) curvilinear velocity, (c) average path velocity (d) straight line velocity (e) linearity and (f) progression. There was a significant improvement in total motility after the addition of 30 μg wild type EV^100K^ (a), but none of the other motility defects were corrected. n = 5–11, mean ± SD, significance compared to *Arrdc4^+/+^* sperm, * *P* < 0.05, ** *P* < 0.01, *** *P* < 0.001, ns = not significant

Since tissue morphology and sperm production appeared normal, we investigated whether there were functional defects within the spermatozoa. Using CASA, motility parameters of sperm harvested from the cauda epididymis were measured. *Arrdc4^–/–^* sperm showed a significant reduction in total motility, curvilinear velocity, average path velocity, straight line velocity, linearity, and progressive motility compared to wild type mice (Figure 4).

To determine the importance of EVs in the sperm motility defect, *Arrdc4^–/–^* sperm were incubated with EV^100K^ harvested from wild type EECs. EV^100K^ were labelled with the green fluorescent general cell membrane dye PKH67 to track uptake by sperm. Sperm have been previously shown to take up EVs in as little as 5 min (Zhou et al., 2019). Both wild type and *Arrdc4^–/–^* sperm were able to take up PKH67‐labelled EVs after 15 min incubation, which localized primarily to the post‐acrosomal region of the head, and the tail midpiece (Figure S4a–c). *Arrdc4^–/–^* sperm were also able to incorporate EV proteins, as shown by an increase in levels of pyruvate kinase M (PKM; Figure S4d, e). Incubation of wild type EV^100K^ with *Arrdc4^–/–^* sperm somewhat improved the total number of motile sperm (Figure 4a) but not the specific motility characteristic parameters (Figure 4b–f). Since there appeared to be no obvious morphological changes in sperm tail architecture to explain the reduced motility in *Arrdc4^–/–^* sperm, we measured mitochondrial function to determine whether energy production may be affected. Mitochondrial membrane potential and mitochondrial reactive oxygen species were not significantly different between any of the groups (Figure S5) suggesting that mitochondrial function is not impaired.

**FIGURE 5:**
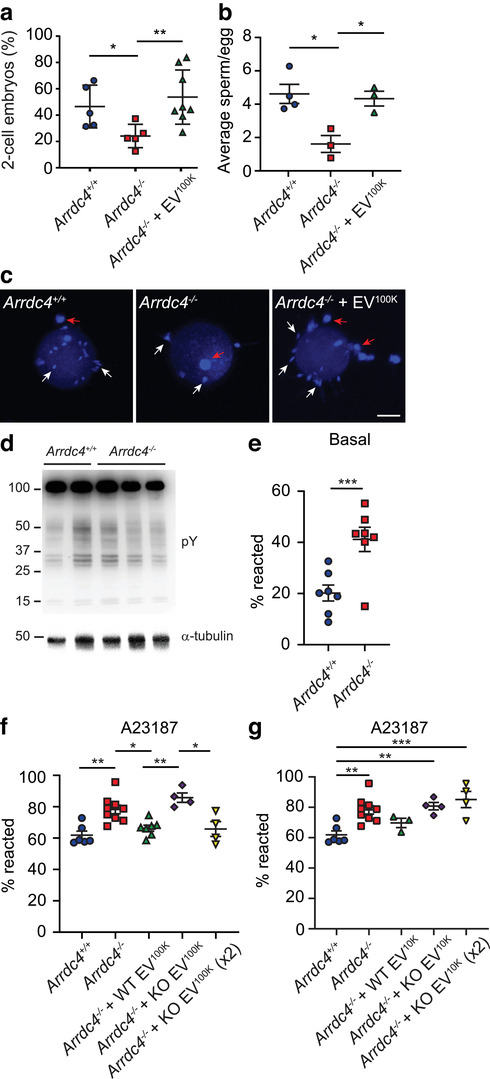
Sperm from *Arrdc4^–/–^* mice have impaired fertilization capabilities which is rescued by wild type EVs. (a) *Arrdc4^–/–^* sperm have reduced ability to fertilize oocytes, as demonstrated by the reduction in the production of two‐cell embryos after overnight incubation. This defect was fully rescued by the addition of 30 μg wild type EV^100K^ to the sperm. *n* = 9–11, mean ± SEM, * *P* < 0.05. (B, C) *Arrdc4^–/–^* sperm have reduced ability to bind to the zona pellucida, and this was rescued by the addition of 30 μg wild type EV^100K^. (b) Graphical representation of the average number of sperm bound to oocytes per male (3‐8 oocytes counted per male). n = 3–4, mean ± SEM, * *P* < 0.05. (c) Oocytes were fixed 4 h after fertilization and stained with DAPI to visualize attached sperm nuclei (white arrows). Some cumulus cells are still present (red arrows). Scale bar, 20 μm. (d) *Arrdc4^–/–^* sperm undergo capacitation as indicated by immunoblotting for tyrosine phosphorylated proteins (pY). α‐tubulin was used as a loading control. (e‐g) *Arrdc4^–/–^* sperm undergo premature acrosome reaction and are more sensitive to acrosome reaction stimulation by calcium ionophore (A23187). (e) Basal levels of acrosome reacted sperm measured immediately after harvest was significantly higher in *Arrdc4^–/–^* sperm than wild type sperm. (f) The acrosome reaction induced by calcium ionophore (A23187) was dampened back to wild type levels with the addition of 30 μg wild type EV^100K^ to *Arrdc4^–/–^* sperm. The addition of *Arrdc4^–/–^* EV^100K^ harvested from the same number of cells did not reduce the number of acrosome reacted sperm, but the addition of *Arrdc4^–/–^* EV^100K^ harvested from twice as many cells (KO EV^100K^ x2) was able to dampen the acrosome reaction in *Arrdc4^–/–^* sperm. (g) The addition of wild type large EV fraction only (EV^10K^) had the same effect on calcium ionophore (A23187) induced acrosome reaction as the EV^100K^ fraction, however *Arrdc4^–/–^* EV^10K^ were unable to dampen the acrosome reaction even when harvested from twice as many cells (KO EV^10K^ x2). n = 4–8, mean ± SD, * *P* < 0.05, ** *P* < 0.01, *** *P* < 0.001. Results for wild type and *Arrdc4^–/–^* sperm untreated are the same in Figure 5f and 5g

To assess fertilizing capabilities, cumulus‐oocyte complexes were isolated from wild type female mice (CBAB6F1) and incubated with wild type or *Arrdc4^–/–^* sperm (1000 sperm per oocyte). *Arrdc4^–/–^* sperm showed a reduced capacity to fertilize oocytes *in vitro* as indicated by a reduction in the number of two‐cell embryos generated after overnight incubation (Figure 5a). When sperm were incubated with wild type EV^100K^ prior to IVF the production of two‐cell embryos was restored (Figure 5a). To analyze this in more detail, we investigated the fertilization process and early embryonic development. *Arrdc4^–/–^* sperm incubated with oocytes for 4 h and stained with DAPI showed a reduction in the number of sperm binding to the zona pellucida which was restored with the addition of wild type EV^100K^ to sperm (Figure 5b, c). To enable efficient binding of sperm to the zona pellucida, sperm must be able to capacitate and undergo the acrosome reaction. Capacitation can be measured by the presence of protein tyrosine phosphorylation. *Arrdc4^–/–^* sperm were capacitated to a similar degree as wild type sperm as indicated by similar levels of protein tyrosine phosphorylation between wild type and *Arrdc4^–/–^* samples following 60 min incubation in fertilization medium (Figure 5d). To assess the acrosome reaction, freshly isolated cauda sperm were assayed immediately after collection (basal; Figure 5e) or incubated for 1 h with the calcium ionophore A23187 (Bromfield et al., 2016; Murdica et al., 2020; Navarrete et al., 2016) to induce the acrosome reaction (Figure 5f, g). *Arrdc4^–/–^* sperm showed a significantly higher level of acrosome‐reacted sperm in the basal samples than wild type (Figure 5e) and were more sensitive to A23187 as indicated by an increase in the number of acrosome‐reacted sperm in the A23187 treated samples (Figure 5f, g). The addition of wild type EV^100K^ to *Arrdc4^–/–^* sperm during A23187 treatment was able to reduce the number of acrosome‐reacted sperm back to levels seen in A23187‐treated wild type sperm (Figure 5f).

To determine if the phenotype rescue can be attributed specifically to Arrdc4‐dependent EVs, we treated sperm with EV^100K^ harvested from the same number of *Arrdc4^–/–^* EECs as wild type. *Arrdc4^–/–^* EV^100K^ were unable to restore acrosome‐reacted sperm to the level of wild type sperm (Figure 5f). Given that Arrdc4 is important for EV biogenesis rather than cargo loading (Figure 3), we added *Arrdc4^–/‐^* EV^100K^ harvested from twice as many EEC cells to sperm (KO EV^100K^ x2). By increasing the amount of *Arrdc4^–/‐^* EV^100K^, we were able to restore acrosome‐reacted sperm to the level of wild type sperm. Based on the results from Figure 3, we further hypothesized that Arrdc4 is important for the biogenesis of the larger sized EV fraction, and that it is this fraction that is primarily responsible for sperm function. We added the large EV fraction only (EV^10K^) to sperm to determine whether this fraction could also restore the phenotype. Wild type EV^10K^ were able to dampen the acrosome reactivity of *Arrdc4^–/‐^* sperm in a similar manner to wild type whole EVs, however *Arrdc4^–/‐^* EV^10K^ were not (Figure 5g). Even after adding *Arrdc4^–/‐^* EV^10K^ from an increased number of cells, they were still unable to dampen the reactivity.

### 3.5 Development of blastocysts is partially restored by using sperm treated with EVs

**FIGURE 6:**
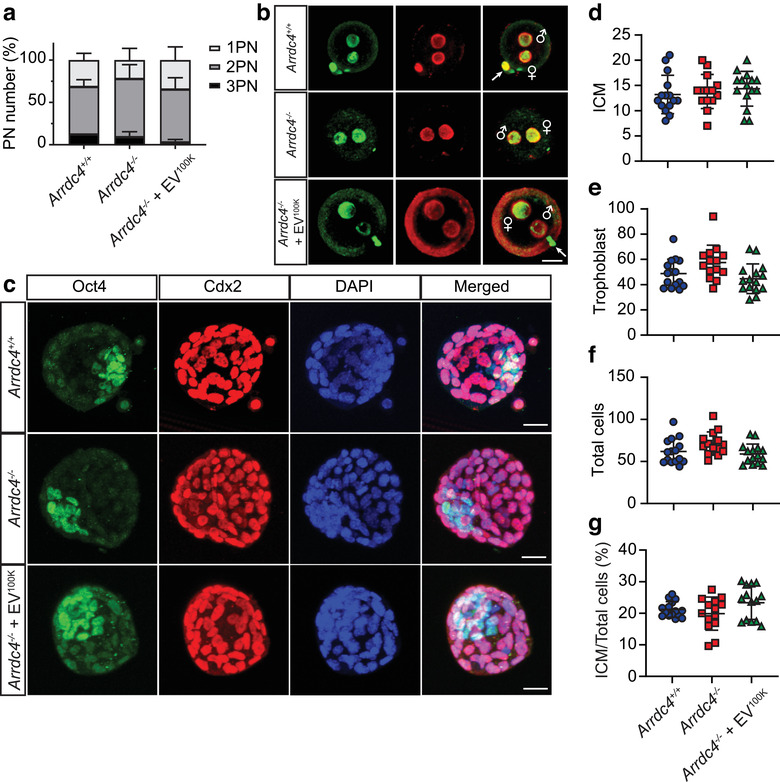
Development of blastocysts generated by *Arrdc4^–/–^* sperm treated with wild type EVs is normal. (a, b) There is no effect of EV^100K^ treatment on the formation of two pronuclei in fertilized oocytes. (a) Percentage of embryos that had 1, 2 or 3 observable pronuclei (PN) in the zygote stage observed via time lapse imaging (TLI) were similar between the groups. n = 4, mean ± SE. (b) Zygotes were fixed 7 h after fertilization and immunostained with 5‐mC (green) and 5‐hmC (red). Both the male and female pronuclei stain with both antibodies as expected at this stage (PN2‐3). Scale bar, 20 μm. (c‐g) Blastocyst cell numbers are not affected by EV^100K^ treatment of *Arrdc4^–/–^* sperm. (c) Immunostaining of blastocysts 103 h after fertilization with Oct4 (inner cell mass, green), Cdx2 (trophoblasts, red) and DAPI (nuclear counterstain, blue). Scale bar, 20 μm. There was no significant difference between groups in the number of (d) inner cell mass cells (ICM), (e) trophoblast cells or (f) total cells in blastocysts. (g) There was also no significant difference in the ratio of ICM to total cells. *n* = 14–15 blastocysts per group fertilized by four males per group, mean ± SD

Because of the beneficial effects of wild type EVs on sperm motility and fertilization, we examined whether these treated sperm could support normal embryo development. To overcome the deficiency in zona‐binding of *Arrdc4^–/–^* sperm, IVF of oocytes from wild type female mice was conducted using a 10‐fold increase in sperm concentration as was used in the previous IVF studies. In this context, we observed no significant difference in the percentage of fertilized zygotes demonstrating the presence of two pronuclei (Figure 6a), and the methylation status of the male and female pronuclei appeared normal as visualized by immunostaining with 5‐mC and 5‐hmC antibodies 7 h after fertilization (Figure 6b). Embryo morphokinetics were measured in a subpopulation of eight presumed zygotes for each male using time‐lapse imaging (TLI) from 4 to 96 h post‐fertilization. There were no perceptible differences between embryos derived from wild type and *Arrdc4^–/–^* sperm (Figure S6) with the exception of increased blastocyst collapse with *Arrdc4^–/–^* sperm and interestingly this was resolved when the sperm were treated with wild type EV^100K^ (Figure S6E, F). Differentiation of late stage blastocysts was assessed by immunostaining 103 h after fertilization with Oct4 (marker of inner cell mass, ICM) and Cdx2 (marker of trophoblasts) (Figure 6c). The number of ICM and trophoblast cells was similar between the groups (Figure 6d, e), and there was no difference between the total number of cells per blastocyst, or the ratio of ICM to total cells (Figure 6f, g). Overall, this data suggests that the addition of exogenous EVs to sperm is compatible with normal embryogenesis and, by increasing cleavage rates and minimizing blastocyst collapse, may be beneficial to embryo quality.

## 4 DISCUSSION

We previously established that Arrdc4 is required for EV biogenesis (Mackenzie et al., 2016), and here show that this cellular process is essential in the epididymis and required for optimal sperm fertilization capacity. Specifically, we show that Arrdc4 is required for the biogenesis of larger particles (> 200 nm) often attributed to direct plasma membrane budding. This is supported by our previous work showing Arrdc4 is required for the biogenesis of particles derived from the plasma membrane in other cell types (Mackenzie et al., 2016). Further, this study shows that Arrdc4‐mediated EV production from EECs is required for normal male reproduction. *Arrdc4^–/‐^* male mice exhibit sub‐fertility attributable to a lack of maturation through the epididymis, caused by reduced EV release from the EECs into the epididymal lumen. Importantly, when *Arrdc4^–/‐^* sperm are supplemented with fractions enriched in EVs from wild type epididymal cells their ability to undergo the acrosome reaction, bind to the zona pellucida and generate two‐cell embryos *in vitro* is restored. *Arrdc4^–/‐^* sperm supplemented with an EV‐enriched fraction from an increased number of *Arrdc4^–/‐^* EECs can also restore the acrosome reaction, indicating that EV deficiency is indeed the underlying reason for sperm dysfunction in these mice and that EVs may represent a new modality for infertility therapy.

The mechanism by which EVs function to assist the fertilization process likely involves preventing premature acrosome reaction. During the acrosome reaction, acrosomal enzymes are released which enable the sperm to dissolve through the zona pellucida and fertilize the egg (Cuasnicú et al., 2016). The sperm must undergo the acrosome reaction at the surface of the zona pellucida, otherwise a premature acrosome reaction leads to the loss of zona pellucida recognition sites from the surface of the sperm, compromising sperm–zona pellucida binding and inhibiting fertilization (Liu & Baker, 1992). EVs from the male reproductive tract have been suggested to inhibit premature acrosome reaction (Arienti et al., 1998; Pons‐Rejraji et al., 2011), however the mechanisms controlling their production and release were unknown. Our data demonstrates that Arrdc4 is a key regulator of the biogenesis of a subset of EVs by EECs and their release into the reproductive tract, thereby suppressing premature acrosome reaction. This finding is in line with the incomplete penetrance of the observed infertility phenotype, with a small proportion of sperm from *Arrdc4^–/–^* mice still having an intact acrosome, enabling these cells to fertilize an egg, albeit at a lower efficiency. Another possibility for the incomplete penetrance of the reproductive phenotype in *Arrdc4^–/–^* mice may be attributable to compensatory mechanisms. Others have shown that mutant alleles produce a milder phenotype than predicted from knockdown studies due to beneficial increases in similar or related genes (Rossi et al., 2015). Indeed, we have seen a significant increase in the mRNA expression of the related gene *Arrdc1* in the cauda epididymis (Figure S7), suggesting that this may be the case.

Our proteomics analysis revealed Arrdc4 regulates several proteins involved in the acrosome vesicle (GO: 0001669, highlighted in red in Figure 2h), cilium movement (GO:0003341, examples highlighted in blue in Figure 2) and sperm motility (GO:0097722, examples highlighted in green) as the sperm travel through the epididymis. Many of these have a demonstrated role in sperm functions such as supressing the acrosome reaction and maintaining acrosome integrity, zona pellucida and oocyte binding, and sperm hyperactivation, capacitation and the acrosome reaction (Carlson et al., 2003; Herrero et al., 2005; Ma et al., 2013; Okunade et al., 2004, Ou *et al*, 2012, Ren et al., 2001, Schuh et al., 2004, Shen et al, 2013). Reduction of these proteins in *Arrdc4^–/–^* cauda sperm could potentially explain the decrease in motility, the thinning of the acrosomal caps and loss of acrosome integrity observed in the *Arrdc4^–/–^* sperm resulting in the reduction in fertility we see in these mice; however, further work is required to confirm this. Proteomics analysis of EV content revealed a similar profile between wild type and *Arrdc4^–/‐^* EV‐enriched fractions, yet NTA, TEM, and immunoblotting clearly demonstrate a reduction in particle number particularly a loss of the larger particle subset (> 200 nm) (Figure 3). Since the addition of more *Arrdc4^–/–^* EV^100K^ to sperm were able to restore the acrosome reaction in a manner similar to wild type EV‐enriched fraction (Figure 5), we conclude that it is the reduction in EV production, and not a change in the trafficking of specific protein cargoes, that is responsible for the observed phenotype in the *Arrdc4^–/–^* mice. When EVs were subfractionated to obtain only the larger sized EVs (using a 10,000 g ultracentrifugation step; EV^10K^), *Arrdc4^–/‐^* EV^10K^ were then unable to rescue the acrosome reaction phenotype, even with a doubling of the quantity (Figure 5), suggesting that both the small and large EVs contribute to the maintenance of the acrosome. Together with the previous data, this indicates that EVs produced by the epididymis are important for preventing premature acrosome reaction, and that reduced EV biogenesis in *Arrdc4^–/–^* mice leads to loss of this function, resulting in inefficient zona pellucida binding by the sperm and a subsequent reduction in fertilization and embryo production.

Several other affected proteins have known roles in maintaining flagellar structure (e.g., Dnah8, Dnah17, Eno4, Spem1). *Dnah17^–/–^* and *Eno4^–/–^* knockout mice demonstrate male infertility due to abnormal development of the axoneme and flagellar structures (Nakamura et al., 2013; Whitfield et al., 2019), while loss of Spem1 in *Spem1^–/–^* mice leads to sperm deformation and loss of motility due to aberrant removal of the cytoplasm during development and retention of excess residual cytoplasm in the cytoplasmic droplet (Zheng et al., 2007). *Arrdc4^–/–^* sperm, while not demonstrating the severity of morphological abnormalities seen in *Dnah17^–/–^*, *Eno4^–/–^* or *Spem1^–/–^* sperm, do show abnormalities in the contents of the cytoplasmic droplet (Figure 2). The function of the cytoplasmic droplet is still poorly described, but it has been suggested that it may assist sperm with maintaining osmolality (Chen et al, 2011; Fetic et al., 2006; Yeung et al., 1999) or that it may serve as an additional energy source during sperm maturation (Yuan et al., 2013), and the presence of the cytoplasmic droplet has been positively correlated with sperm motility (Xu et al., 2013). Whether the abnormal appearance of the cytoplasmic droplet in *Arrdc4^–/–^* sperm inhibits its role in these functions is yet to be determined.

The addition of a wild type EV‐enriched fraction did not completely normalize sperm motility as readily as fertilization capacity. It is possible that *Arrdc4^–/–^* sperm have subtle structural differences (due to alterations in flagellar proteins described above) that cannot be repaired by the addition of our EV fractions, or that other non‐EV constituents of the epididymal milieu such as soluble proteins or metabolites are also required. Arrdc4 has been shown to be important for glucose transport and insulin signalling (Ahn et al., 2016; Dagdeviren et al, 2020; Patwari et al, 2009; Richards et al., 2018; Wilde et al., 2019), and thus it may be through this role that Arrdc4 can control epididymal metabolite content. Further analysis into the composition of epididymal luminal fluid from these mice may reveal additional changes that could explain why the EV‐enriched fractions were unable to fully rescue the motility defects.

Interestingly, treatment of sperm with the EV‐enriched fractions showed an intriguing effect on subsequent blastocyst development (Figure S6). During hatching from the zona pellucida, blastocysts undergo weak contractions and pulsations, however a stronger collapse is correlated with poor implantation success (Marcos et al, 2015; Niimura, 2003; Sciorio et al, 2020). The decrease in the incidence and number of collapses in blastocysts from the EV^100K^ treated sperm suggests they may be beneficial to embryo quality.

In generating the *Arrdc4^–/–^* mice, we have produced a mouse model of male sub‐fertility, the cause of which could not be detected using standard clinical methods (WHO standard semen analysis and tissue morphology), and which was normalized by treatment with EV‐enriched fractions. Thus, disruptions to EV production may be an underlying cause of a subset of idiopathic male infertility. Importantly, our study elucidates a cellular mechanism by which EV production is regulated *in vivo* and which is essential for male reproduction capacity.

## CONFLICT OF INTEREST

The authors declare no competing interests.

## AUTHOR CONTRIBUTIONS

Natalie J. Foot, Macarena Gonzalez, Rebecca L. Robker and Sharad Kumar designed the project. Natalie J. Foot, Macarena Gonzalez, Kelly Gembus, Diana Tran, Pamali Fonseka and Jarrod J. Sandow performed experiments. Natalie J. Foot, Macarena Gonzalez, Jarrod J. Sandow, Andrew I. Webb, Rebecca L. Robker, Sharad Kumar and Kelly Gembus analysed data and contributed to manuscript writing and editing. Kelly Gembus and Sharad Kumar acquired funding.

## Supporting information

Supporting information.

Supporting information.

Supporting information.

Supporting information.

Supporting information.

Supporting information.

Supporting information.

Supporting information.

Supporting information.

Supporting information.

Supporting information.

Supporting information.

Supporting information.
