# Erratum: Arrdc4‐dependent extracellular vesicle biogenesis is required for sperm maturation

**DOI:** 10.1002/jev2.12126

**Published:** 2021-07-24

Foot, N. J., Gonzalez, M., Gembus, K., Fonseka, P., Sandow, J. J., Nguyen, T. T., Tran, D.,Webb, A. I., Mathivanan, S., Robker, R. L., & Kumar, S. (2021). Arrdc4‐dependent extracellular vesicle biogenesis is required for sperm maturation. *Journal of Extracellular Vesicles*, **10**(8), e12113. https://doi.org/10.1002/jev2.12113

Suresh Mathivanan's name was incorrectly replaced with Kelly Gembus’ name in two places in the Author Contributions section of the article. The correct text is:

Natalie J. Foot, Macarena Gonzalez, Jarrod J. Sandow, Andrew I. Webb, Rebecca L. Robker, Sharad Kumar and Suresh Mathivanan analysed data and contributed to manuscript writing and editing. Suresh Mathivanan and Sharad Kumar acquired funding.

