# Supplementary material for: Arrdc4‐dependent extracellular vesicle biogenesis is required for sperm maturation
Source: J Extracell Vesicles. 2021 Jun 22;10(8):e12113. doi: 10.1002/jev2.12113 (PMC8217992; doi:10.1002/jev2.12113)

Figure S1

A

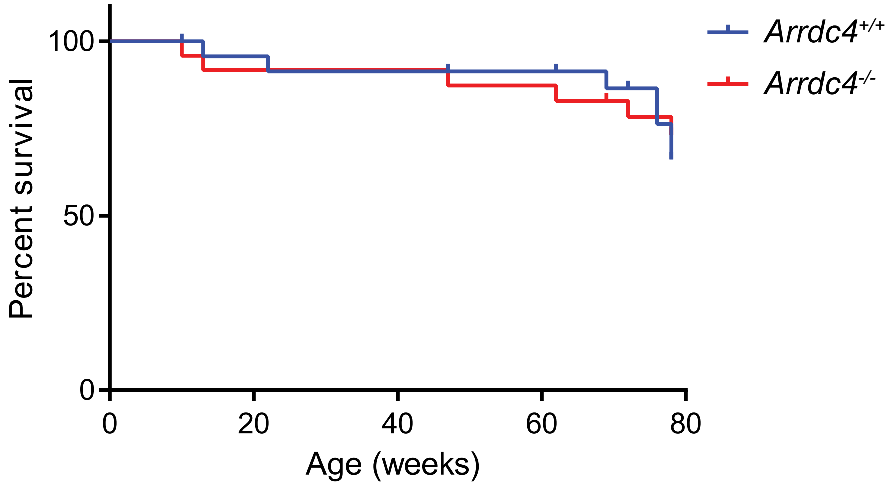

B

Cause of death	*Arrdc4*+/+	*Arrdc4*-/-
Skin wounds due to overgrooming	3	1
Ovarian cyst	1	
Eye abscess	1	
Fighting injury	1	
Hydrocephalus	1	
Hydronephrosis		1
Infection		2
Tumour	2	1
Unknown*		2

C

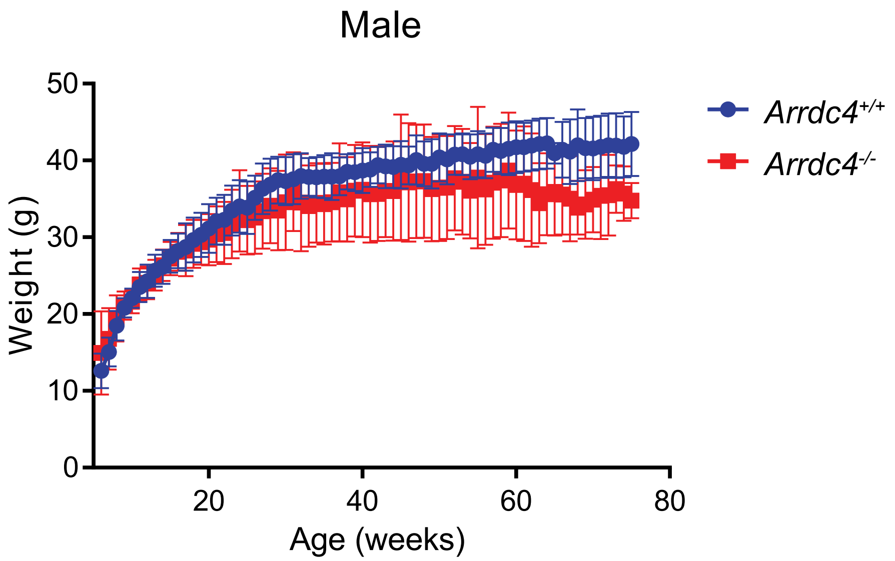

D

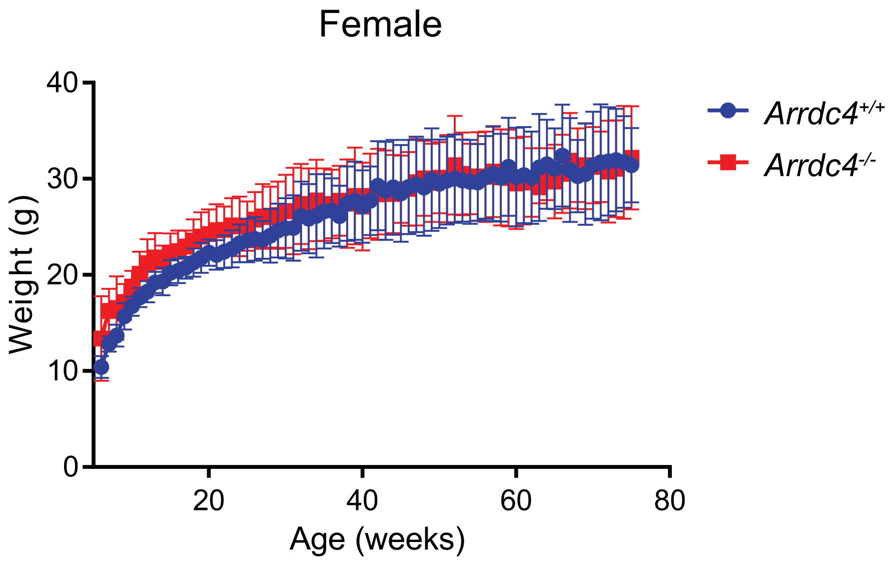

Supplement: Supplementary file 1 — Supporting information. [file JEV2-10-e12113-s002.pdf]
