# Supplementary figures and images for: Arrdc4‐dependent extracellular vesicle biogenesis is required for sperm maturation
Source: J Extracell Vesicles. 2021 Jun 22;10(8):e12113. doi: 10.1002/jev2.12113 (PMC8217992; doi:10.1002/jev2.12113)

Figure S2

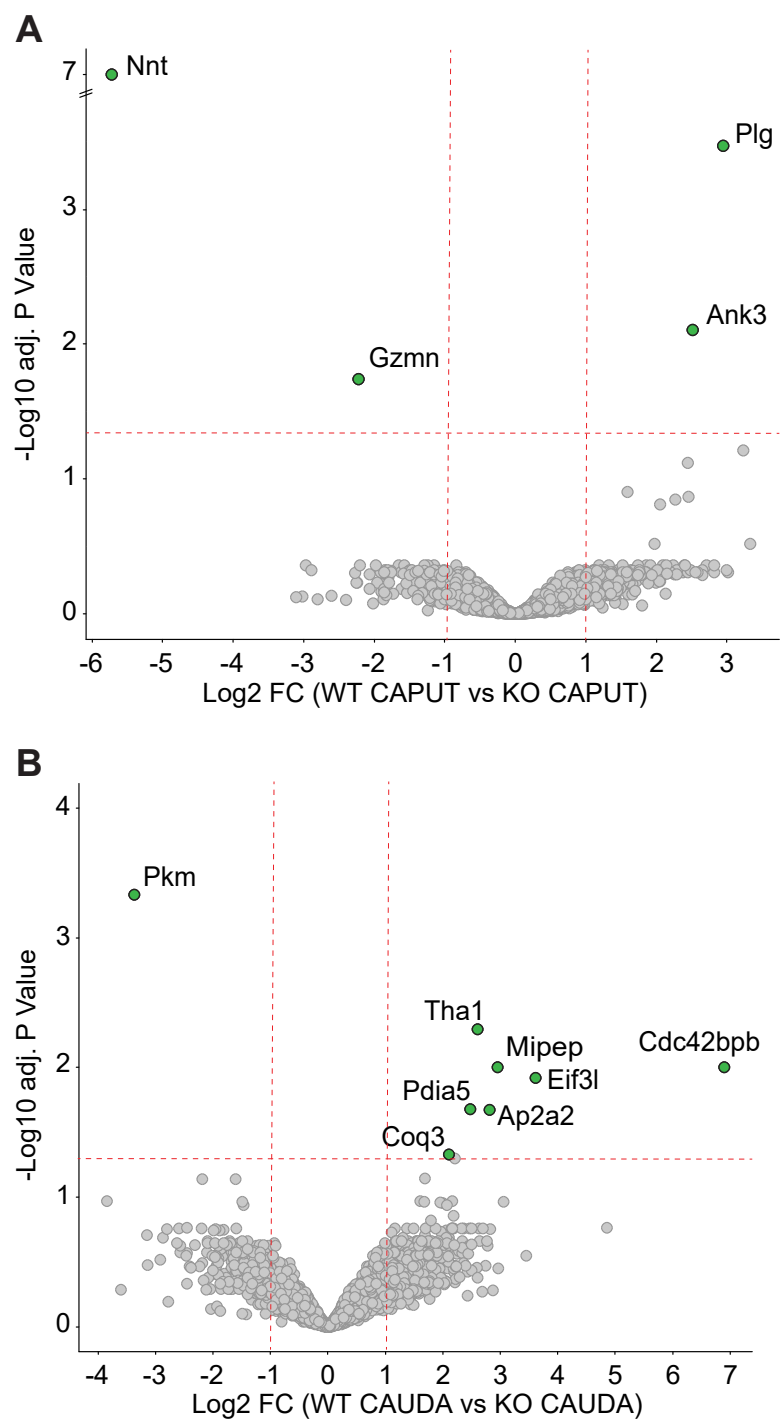

Supplement: Supplementary file 2 — Supporting information. [file JEV2-10-e12113-s001.pdf]

Figure S3

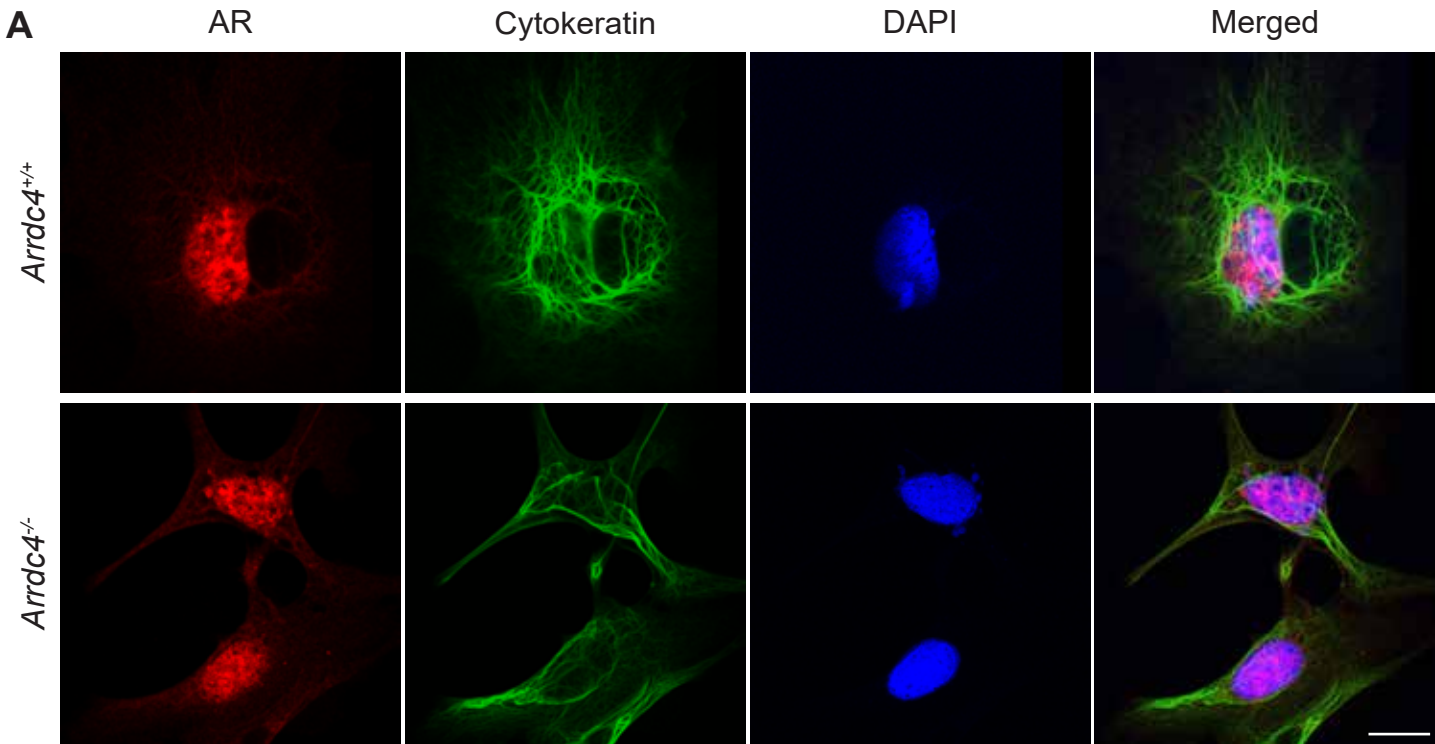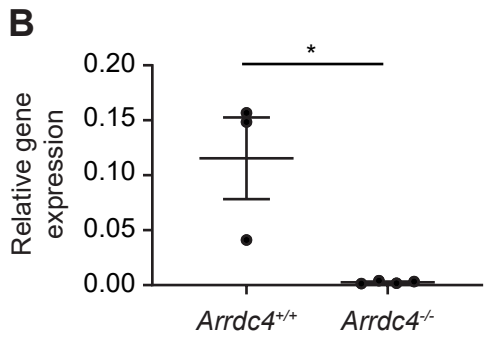

Supplement: Supplementary file 3 — Supporting information. [file JEV2-10-e12113-s010.pdf]

Figure S4

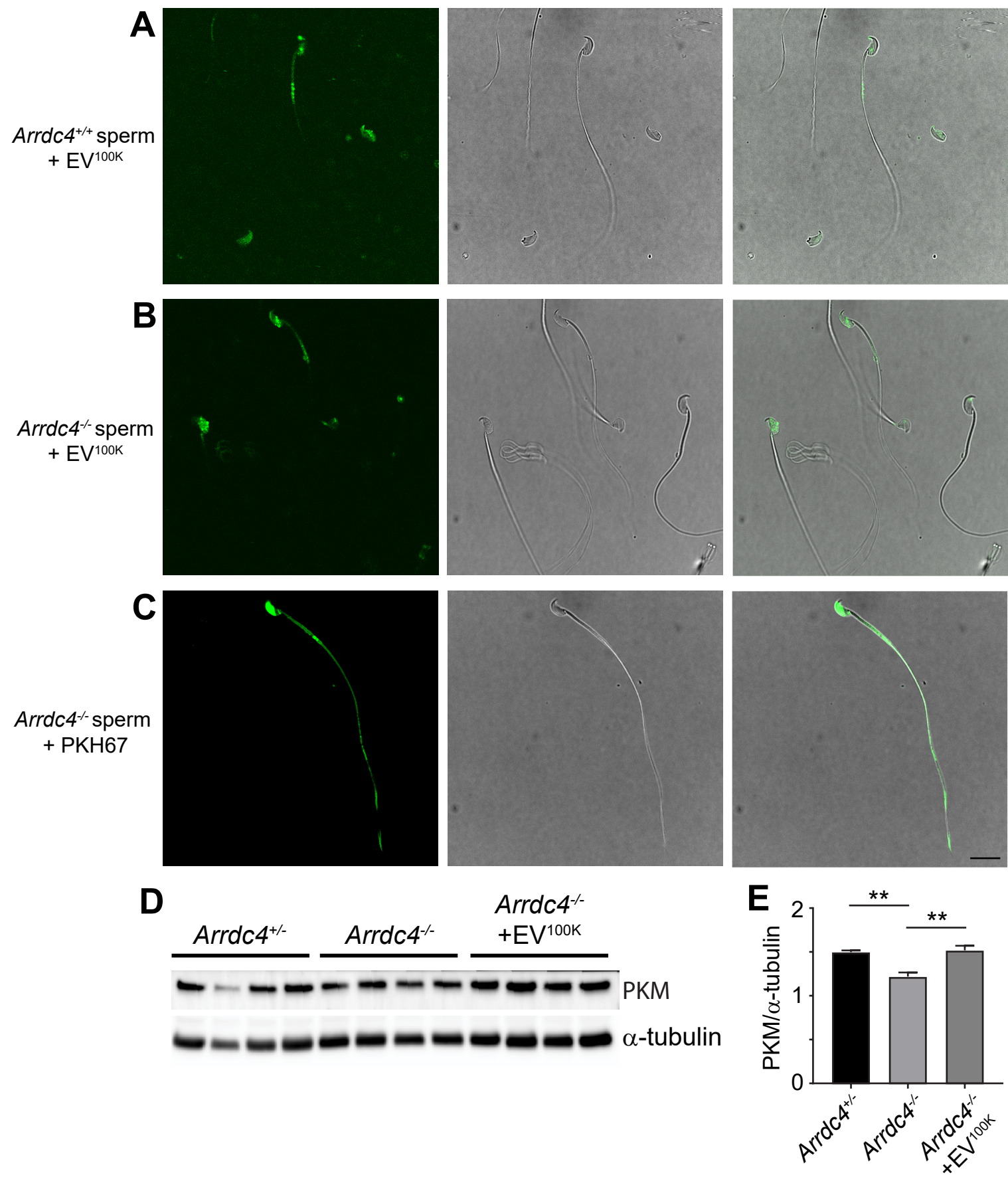

Supplement: Supplementary file 4 — Supporting information. [file JEV2-10-e12113-s012.pdf]

Figure S5

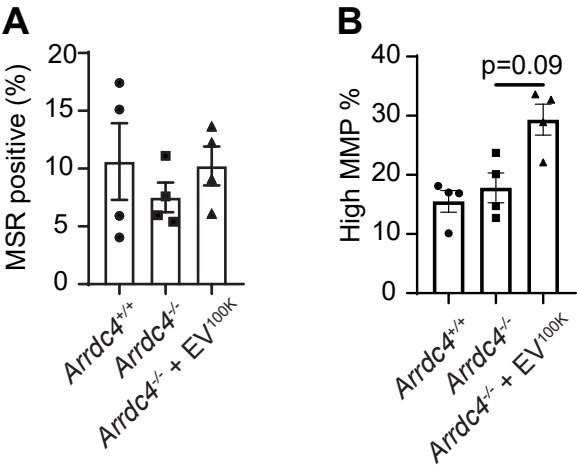

Supplement: Supplementary file 5 — Supporting information. [file JEV2-10-e12113-s005.pdf]

Figure S6

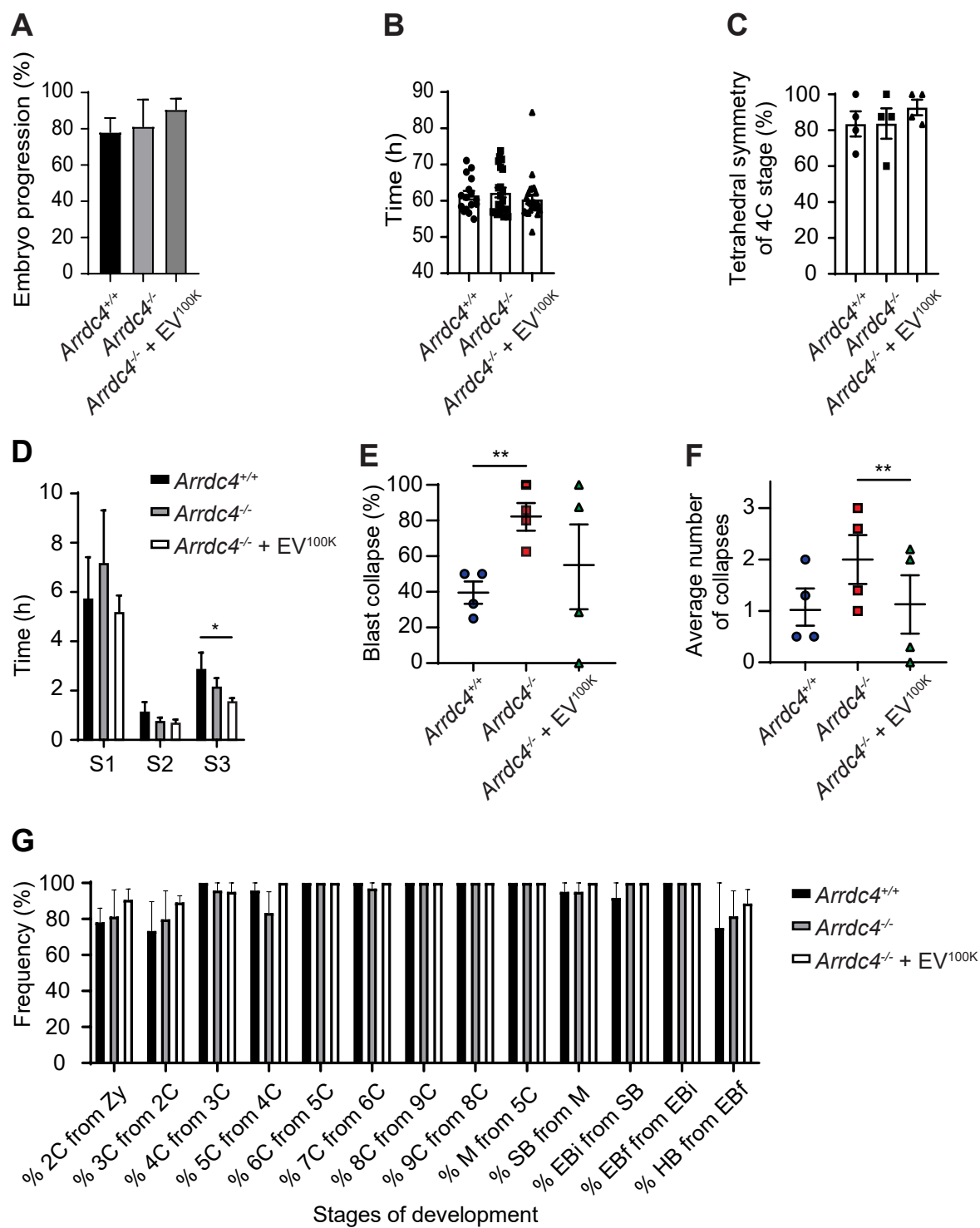

Supplement: Supplementary file 6 — Supporting information. [file JEV2-10-e12113-s007.pdf]

Figure S7

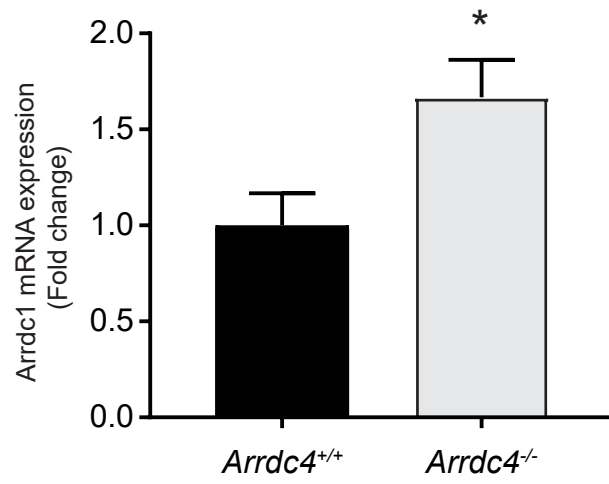

Supplement: Supplementary file 7 — Supporting information. [file JEV2-10-e12113-s013.pdf]
