# Supplementary material for: Arrdc4‐dependent extracellular vesicle biogenesis is required for sperm maturation
Source: J Extracell Vesicles. 2021 Jun 22;10(8):e12113. doi: 10.1002/jev2.12113 (PMC8217992; doi:10.1002/jev2.12113)
Supplement: Supplementary file 8 — Supporting information. [file JEV2-10-e12113-s003.docx]

**SUPPLEMENTAL INFORMATION**

**Figure S1. Phenotypic observations in *Arrdc4^-/-^* mice.**

(A) Kaplan-Meier survival curves for wild type and *Arrdc4^-/-^* mice showing no significant differences in survival. Both males and females are included in the curve. n = 24.

(B) Causes of death for mice used for observing survival rates. No cause of death was associated with genotype. * Clinical signs of hunched posture, ruffled fur and reluctance to move were observed, but no obvious cause was found.

(C, D) Growth curves for (C) male and (D) female mice. There is no significant difference in growth rates between wild type and *Arrdc4^-/-^* mice in males or females. Mean ± SD, n=24.

**Figure S2. Differential protein expression in sperm.**

(A) Volcano plots demonstrating proteins significantly changed by greater than two-fold and a P value < 0.05 (dotted line) in wild type compared with *Arrdc4^-/-^* sperm isolated from the caput epididymis.

(B) Volcano plots demonstrating proteins significantly changed by greater than two-fold and a P value < 0.05 (dotted line) in wild type compared with *Arrdc4^-/-^* sperm isolated from the cauda epididymis. Significantly changed proteins have been highlighted in green.

**Figure S3. Verification of epididymal epithelial cells (EECs).**

(A) EECs were immunostained with antibodies for Androgen Receptor (AR, red) and cytokeratin (epithelial marker, green), and DAPI (nuclear marker, blue). Scale bar, 15 µm.

(B) qPCR confirmed knockout of Arrdc4 gene expression. Mean ± SE, n=3, * p<0.05.

**Figure S4. PKH67-labelled EVs target to sperm head and midpiece.**

(A, B) Green fluorescence indicating uptake of labelled EV^100K^ is visible in the post-acrosomal region of the head, and in the tail midpiece in both wild type and *Arrdc4^-/-^* sperm.

(C) Sperm labelled with PKH67 dye directly shows fluorescent labelling of whole sperm. This localization differed from sperm treated with PKH67-labelled EV^100K^, confirming that the green fluorescence in sperm was due to EV uptake rather than free unincorporated dye. Scale bar, 10 µm.

(D, E) Immunoblotting and quantitation by ImageJ show PKM levels are decreased in *Arrdc4^-/-^* sperm compared to wild type but are restored upon the addition of 30 µg wild type EV^100K^. Mean ± SE, n = 4, ** p<0.01.

**Figure S5. Mitochondrial function in sperm.**

(A) Percentage of sperm cells presenting high intracellular levels of reactive oxygen species ROS, as measured by Flow Cytometry using MitoSox Red.

(B) Percentage of sperm cells presenting high mitochondrial membrane potential (MMP) as measured by Flow Cytometry using JC-1. EV^100K^ therapy tended to increase MMP in sperm compared to sperm from untreated *Arrdc4^-/-^* males, but this was not statistically significant. n = 4, mean ± SE.

**Figure S6. Early embryo development assessed by morphokinetics and time lapse imagery.**

(A) Embryos with confirmed 2PN progressed through first cleave and the 2C stage as expected. (B) The time from PNf to EBi was similar between the groups.

(C) Embryos that developed to the 4C stage had tetrahedral symmetry as expected.

(D) EV^100K^ treatment accelerated the third round of blastomere mitotic division (S3) in embryos from treated *Arrdc4^-/-^* males compared to embryos from wild type males, but this did not affect overall length of development. S1-3: Time to complete synchronous divisions. S1: Time (h) from PNf (fading of the pronuclei) to 2-cell - first round of synchronous division. S2: Time (h) from 3-cell to 4-cell - second round of synchronous divisions. S3: Time (h) from 5-cell to 8-cell - third round of synchronous divisions.

(E, F) Embryos derived from *Arrdc4^-/-^* sperm had an increased chance of undergoing blastocyst collapse, but EV^100K^ treatment diminished the average number of collapses per embryo to levels similar to embryos from wild type males.

(G) Embryos successfully achieved consecutive stages of development in all groups.

n = 16-28 embryos generated from 4 males for each group, mean ± SE, * p<0.05. ** p<0.01.

Timing of specific events during pre-implantation embryo development or kinetic markers: Zy: zygote stage. 2C-9C: interval, absolute time (h) between the 2-cell (2C) (first mitotic embryonic division), and the 9-cell (9C) stages (eighth mitotic embryonic division). M: morula, end of the compaction stage. SB: appearance of the blastocoel cavity, initiation of blastulation. EBi: Blastocoel reaches 50% of blastocyst volume, initiation of blastocyst expansion. EBf: Blastocoel reaches >90% of blastocyst volume, full blastocyst expansion. HB: initiation of blastocyst hatching out of the zona pellucida.

**Figure S7. Compensatory gene expression in *Arrdc4^-/-^* mice.**

There is a significant increase in *Arrdc1* mRNA expression in the epididymis of *Arrdc4^-/-^* mice. n = 3, mean ± SE, * p < 0.05.

**Supplemental Table S1. Primers used for genotyping and quantitative PCR.**

| Primers |  |
| --- | --- |
| Arrdc1 QPCR F | CCGCGTCGTCGTACGGTC |
| Arrdc1 QPCR R | AGACTTCCCTTGTCAGCCAGT |
| Arrdc4 QPCR F | AGTGTCGCAAGCCAGTTCAG |
| Arrdc4 QPCR R | CTTCCCCGTCACAGTCAGAG |
| TBP QPCR F | CAAACCCAGAATTGTTCTCCTT |
| TBP QPCR R | ATGTGGTCTTCCTGAATCCCT |
| Genotyping P1 | CTGGGGCCACTGCGGATCTTGG |
| Genotyping P2 | CAAGCTCTTGACACGGCCCCGAG |
| Genotyping P3 | GCTGGCTTGGTCTGTCTGTCCTA |

All primers purchased from Integrated DNA Technologies

**Supplemental Table S2. Parameters used by ImageJ in CASA sperm motility analyses.**

| CASA parameter | Value |
| --- | --- |
| Minimum sperm size (pixels) | 5 |
| Maximum sperm size (pixels) | 60 |
| Minimum track length (frames) | 10 |
| Maximum sperm velocity between frames (pixels) | 20 |
| Minimum VSL for motile (um/s) | 3 |
| Minimum VAP for motile (um/s) | 7 |
| Minimum VCL for motile (um/s) | 8 |
| Low VAP speed (um/s) | 2 |
| Maximum percentage of path with zero VAP | 1 |
| Maximum percentage of path with low VAP | 25 |
| LowVAP speed 2 (um/s) | 8 |
| Low VCL speed (um/s) | 12 |
| High WOB (percent VAP/VCL) | 80 |
| High LIN (percent VSL/VAP) | 80 |
| High WOB 2 (percent VAP/VCL) | 50 |
| High LIN 2 (percent VSL/VAP) | 60 |
| Frame rate | 31 |
| Microns per 1000 pixels | 346 |
